# The Prevalence and Prognostic Role of PD-L1 in Upper Tract Urothelial Carcinoma Patients Underwent Radical Nephroureterectomy: A Systematic Review and Meta-Analysis

**DOI:** 10.3389/fonc.2020.01400

**Published:** 2020-08-21

**Authors:** Yi Lu, Jiaqi Kang, Zhiwen Luo, Yuxuan Song, Jia Tian, Zhongjia Li, Xiao Wang, Li Liu, Yongjiao Yang, Xiaoqiang Liu

**Affiliations:** ^1^Department of Urology, Tianjin Medical University General Hospital, Tianjin, China; ^2^Department of Hepatology, National Clinical Research Center for Cancer and Cancer Hospital, Beijing, China; ^3^Department of Urology, The Second Hospital of Tianjin Medical University, Tianjin, China

**Keywords:** PD-L1, immune checkpoint, prognostic biomarker, meta-analysis, upper tract urothelial carcinoma (UTUC)

## Abstract

**Background:** Several studies investigating the role of PD-L1 in upper tract urothelial carcinoma (UTUC) patients after radical nephroureterectomy (RNU) to predict prognosis had been published and great controversy existed among them. We, therefore, in the meta-analysis, reported the association between PD-L1 and survival in UTUC patients who underwent RNU.

**Methods:** We searched the PubMed, Cochrane Library, EMBASE, and Web of Science by April 1, 2020. Hazard ratio (HR) and odds ratio (OR) were adopted to evaluate relationships between PD-L1 and survival outcomes, and tumor features, respectively. We formulated clinical questions and organized following the PICOS strategy.

**Results:** Eight retrospective studies incorporating 1406 patients were included. The pooled positive rate of PD-L1 in UTUC patients was 21.0% (95% CI = 13.0–30.0%, *I*^2^ = 95.3%). Furthermore, higher PD-L1 in tumor tissues was related to shorter cancer-specific survival (CSS) in radically resected UTUC patients (HR = 1.63, 95% CI = 1.17–2.26, *I*^2^ = 0.0%), but was not associated with overall survival (OS) (HR = 1.49, 95% CI = 0.76–2.91, *I*^2^ = 74.9%). Subgroup analyses indicated associations between higher PD-L1 and shorter CSS in both Caucasus (HR = 1.72, 95% CI = 1.02–2.92, *I*^2^ = 0.0%) and Asian (HR = 1.57, 95% CI = 1.03–2.39, *I*^2^ = 23.1%) UTUC patients. Furthermore, PD-L1 was related to tumor grade of UTUC (High vs. Low, OR = 3.56, 95% CI = 1.82–6.97, *P* = 0.000) and invasive depth (pT3+pT4+pT2 vs. pT1+pTa/pTis, OR = 2.53, 95% CI = 1.07–5.96, *P* = 0.001). In the cumulative meta-analysis, results indicated that the 95% CIs narrowed as the pooled results gradually moved near the null.

**Conclusions:** PD-L1 overexpression was related to worse survival outcomes in UTUC patients after RNU. It may be useful to incorporate PD-L1 into prognostic tools to select appropriate treatment strategies for UTUC. PD-L1 can also be clinically used for survival anticipation, risk stratification, and patient counseling. However, the pooled findings should be considered tentative until ascertained by more researches.

## Introduction

Upper tract urothelial carcinoma (UTUC) is a kind of rare transitional cell carcinoma that has a rate of 1/50,000 in developed countries. An overwhelming majority of urothelial carcinomas (UCs) are found in the urinary bladder, whereas only 5–8% were UTUC (1, 2). Currently, the golden treatment for localized UTUC is radical nephroureterectomy (RNU), while many of the patients will suffer recurrence, metastasis, and decreased renal function even after RNU. Furthermore, ~60% of UTUC cases are locally advanced or muscle-invasive at initial diagnosis because of its occult symptoms and delayed diagnosis (1, 3, 4). Based on these dilemmas, there is a pressing need for a novel curable and safe treatment.

Over the past decade, the immune checkpoint and the landmark achievements in tumor researches, which revealed the mechanisms of tumor genesis and development, have been widely discussed (5, 6). Recently, the favorable efficacy of immunotherapy has been validated in many malignancies, including UC of the bladder (7–10). PD-L1, on the tumor cells, could bind to PD-1 and suppresses immune cell proliferation and release of immune factors, such as cytokine, and eventually evades immune surveillance through immune checkpoints to realize tumor recurrence or metastasis (11, 12).

In recent years, studies have increased dramatically in investigating the association between PD-L1 and survival outcomes in radically resected UTUC patients and the conclusion is still controversial. Some evidence indicated that higher PD-L1 was related to poor survival for UTUC (13, 14), while some reported opposing findings (15–17). Therefore, we formulated clinical questions following the PICOS strategy and firstly assessed whether PD-L1 expression (high or low) was related to survival outcomes or the clinicopathological features in UTUC patients after RNU with no restriction to the study designs through a meta-analysis.

## Methods

### Data Sources

We made a detailed inclusion criterion in accordance with the established reporting guidelines before searching the evidence (18, 19). We systematically reviewed all available English language literature in PubMed, Cochrane Library, EMBASE, and Web of Science in April 2020. No eligible randomized controlled trials (RCTs) were found and observational researches that focused on the associations between PD-L1 and tumor behaviors and survival outcomes in UTUC patients after RNU were included. We also searched and checked the references and citations of retrieved articles carefully. Three authors conducted the search process independently. The keywords for the search were “PD-L1,” “urothelial carcinoma,” and “upper tract.” Detailed search strategy and the PICOS tool were summarized in Supplementary Table 1.

### Criteria for Inclusion and Exclusion

Inclusion criteria: (a) Population: Radically resected UTUC patients with or without non-surgical treatments. (b) Interventions: High levels of PD-L1 (≥cutoff value) in tumor tissue. (c) Comparators: Low rate of PD-L1 (<cutoff value) in tumor tissue. (d) Outcomes: Survival outcomes or clinicopathological characteristics of UTUC cases, such as tumor grade, recurrence, etc. (e) Study design: Observational studies (prospective or retrospective) or RCTs. (f) Article types: Original article. (g) Studies in the English language. (h) Information on survival outcomes: Hazard ratio (HR) and 95% confidence interval (95% CI) could be obtained directly or indirectly. (i) Studies with sample size of more than 50 and mean/median follow-up duration of more than 12 months. We excluded those studies that cannot meet the inclusion criteria.

### Data Collection

The retrieved records were screened by the three authors. The details including the first author, study year, study design, study region, demographic information, cutoff value (PD-L1 expression), median follow-up duration, and survival outcomes were recorded from all studies. By contacting the article authors, we obtained both missing and unclear information. When the authors did not reply, information was considered as not available. By using the validated tool (20), we digitized and derived the HRs and their 95% CIs from studies that only had Kaplan–Meier curves showing survival outcomes.

### Risk of Bias (RoB) Assessment

The RoB were evaluated by each of the three authors independently using a modified Newcastle–Ottawa scale (NOS) (21). Agreement in the assessment was reached through consensus among the three authors and communication with article authors.

### Statistical Analysis

Pooled ORs were used to indicate the relationship between PD-L1 expression and features of UTUC cases. We pooled HR to reveal the relationship between PD-L1 and survival outcomes. If significant heterogeneity was found and *I*^2^ > 50%, we utilized random-effect models; otherwise, we selected fixed-effect models (22). We conducted Begg's test, Egger's test, and created funnel plots to assess the publication bias. Sensitivity analyses were done by excluding a study at one time and a cumulative meta-analysis was also done. We performed statistical analyses by using STATA 12.0 (Stata-Corp.). Results were of statistical significance at two-tailed *P* < 0.05.

## Results

### Literature Selection

**Figure 1:**
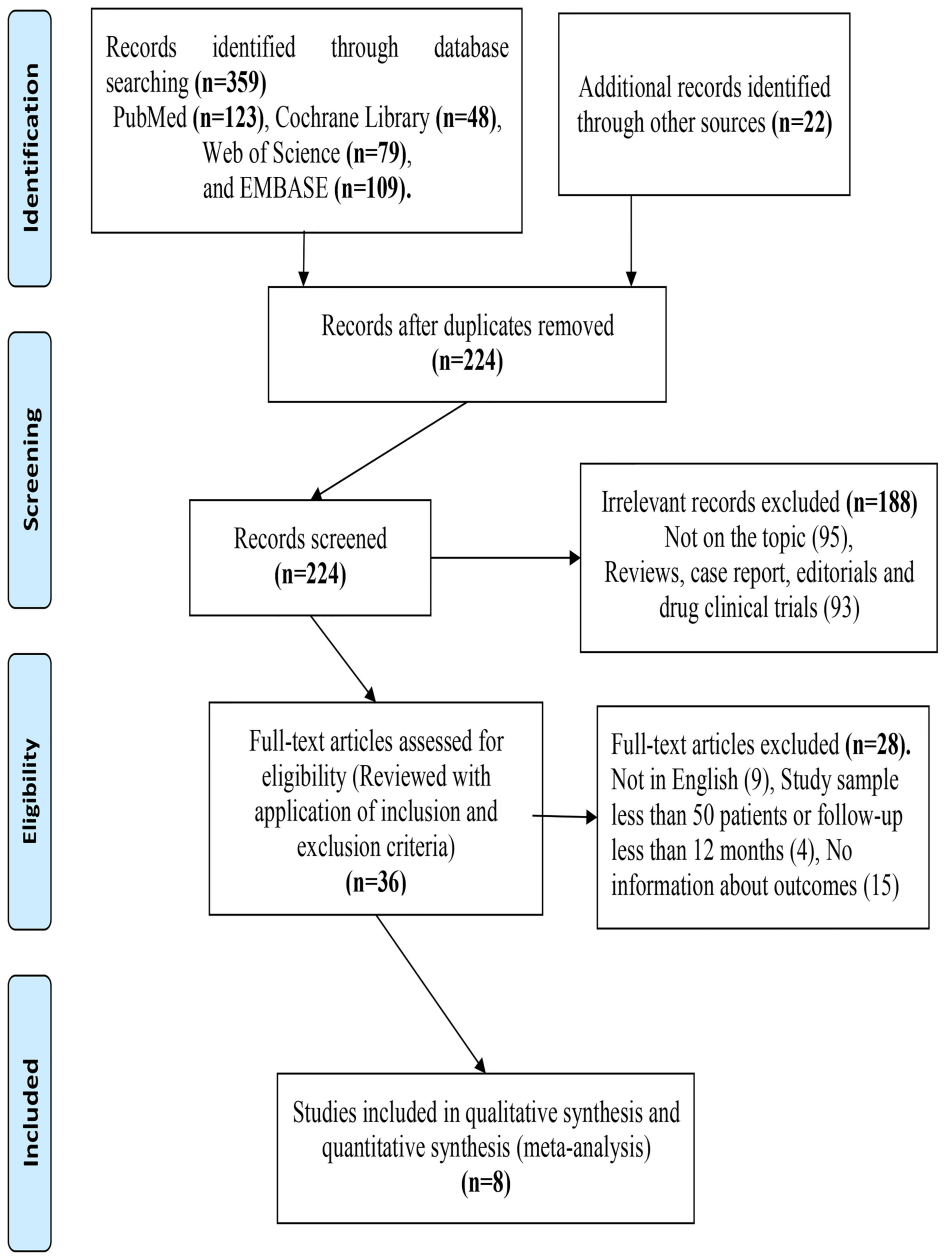
PRISMA flow chart of the data search.

Following the established literature selection strategy above, we identified 224 non-repeated records. We excluded records for the following reasons: article types or not relevant topics (*n* = 188), studies had few study samples (≤50) or follow-up duration less than 12 months (*n* = 4), not in English (*n* = 9), or insufficient survival data (*n* = 15). Finally, we included eight retrospective cohort studies (1,406 individuals) (13–17, 23–25) in the study (shown in Figure 1).

### Characteristics of Included Studies

We included eight retrospective studies, which were published in the recent 4 years. The median follow-up periods among these studies were from 23.0 to 98.7 months (Table 1). A total of 1406 patients were included, with each of them receiving nephroureterectomy. These studies were conducted across two regions, with three in the USA and five in Asia. All the studies adopted immunohistochemistry (IHC) to analyze the level of PD-L1 in tumor tissues. Notably, the intensity of PD-L1 in IHC was only mentioned in four studies (14, 23–25), while none of them reported neither the mean/median intensity nor its linear correlation with the outcomes. The NOS grades indicated that all studies had high qualities (Supplementary Table 2).

**Table 1:** Characteristics of included studies.

**Reference**	**Study type**	**Study region**	**Ethnicity**	**Sample size**	**Treatment**	**Evaluated cells**	**Cutoff value for PD-L1**	**Positive PD-L1 (%)**	**Median follow-up (months)**	**Survival**
Skala et al. (24)	RC	USA	Caucasus	149	RNU: all; AC: 17 (11.4%); NC: 18 (12.1%); immunotherapy: N.R.	Tumor cells (PD-L1)	≥5%	23.5	24.8	CSS
Krabbe et al. (15)	RC	USA	Caucasus	423 (high-grade UTUC)	RNU: all; AC, NC, immunotherapy: none	Tumor-infiltrating lymphocytes (PD-1) and tumor cells (PD-L1)	≥1%	26.2	37.0	OS, CSS, RFS
Zhang et al. (13)	RC	China	Asian	162	RNU: all; AC, NC: N.R. immunotherapy: none	PD-L1 in tumor cells and TIMCs	≥5%	12.3	79.0	CSS
Miyama et al. (16)	RC	Japan	Asian	271	RNU: all; AC, NC: N.R. immunotherapy: N.R.	Circulating platelets and tumor cells (PD-L1)	≥5%	11.0	52.0	OS, MFS
Arriola et al. (23)	RC	USA	Caucasus	72	RNU: all; AC, immunotherapy: N.R.; NC: none	Tumor-infiltrating lymphocytes (PD-1) and tumor cells (PD-L1)	≥1%	37.5	98.7	OS, CSS
Wang et al. (25)	RC	China	Asian	88	RNU: all; AC: 8 (9.1%); NC, immunotherapy: N.R.	Tumor cells (PD-L1/2)	≥1%	23.9	23.0	OS, CSS
Nukui et al. (17)	RC	Japan	Asian	79	RNU: all; AC: 41 (51.9%); NC, immunotherapy: N.R.	Tumor cells and TILs (PD-L1)	≥5%	39.2	45.0	OS, CSS
Kim et al. (14)	RC	Korea	Asian	162 (non-metastatic UTUC)	RNU: all; AC: 72 (44.4%); NC: none; immunotherapy: N.R.	Tumor cells (PD-L1)	≥5%	3.086	53.4	OS, CSS

*RC, retrospective cohort study; RNU, radical nephroureterectomy; AC, adjuvant chemotherapy; NC, neoadjuvant chemotherapy; OS, overall survival; CSS, cancer-specific survival; IHC, immunohistochemistry staining; MFS, metastasis-free survival; RFS, recurrence-free survival; N.R., not reported*.

### Prevalence of PD-L1 in UTUC

**Figure 2:**
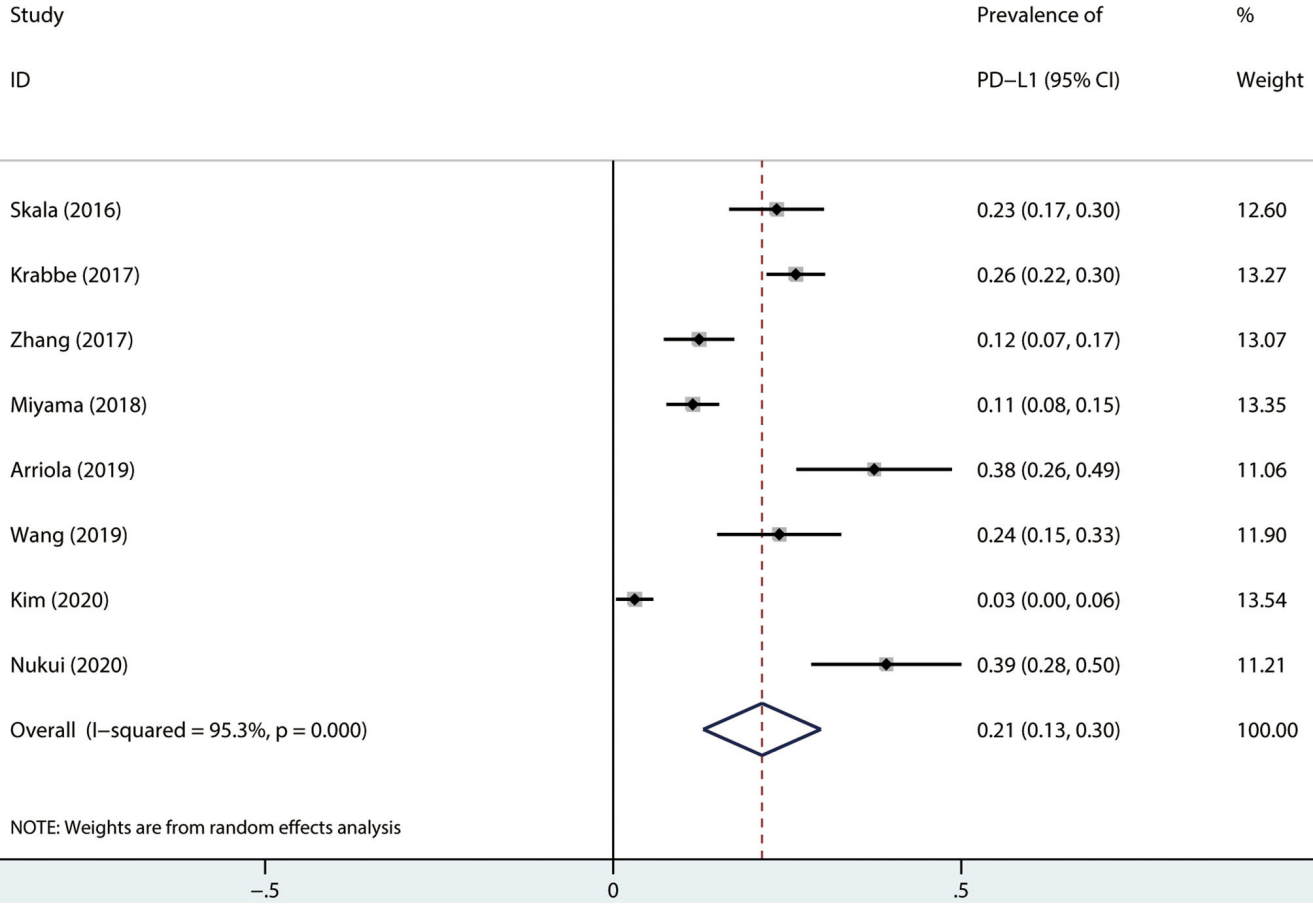
Prevalence of PD-L1 expression in UTUC. CI, confidence interval.

The prevalence range was 3.0–39.2% (Table 1). The pooled prevalence of PD-L1 in UTUC was 21.0% (random effect, 95% CI: 0.13 to 0.30, *I*^2^ = 95.3%; Figure 2).

### Association Between PD-L1 and Survival

**Figure 3:**
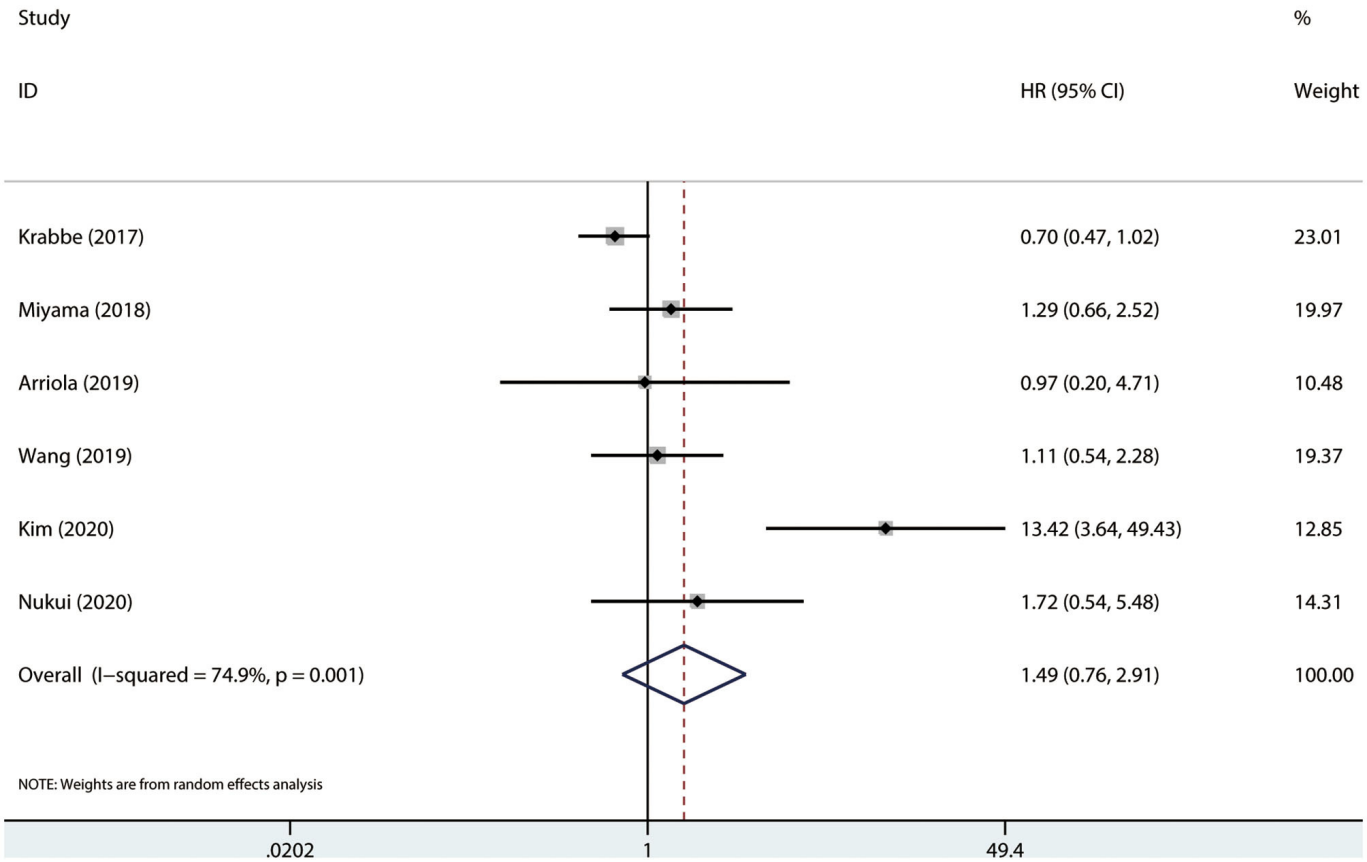
Prognostic value of PD-L1 for OS. OS, overall survival; HR, hazard ratio; CI, confidence interval.

**Figure 4:**
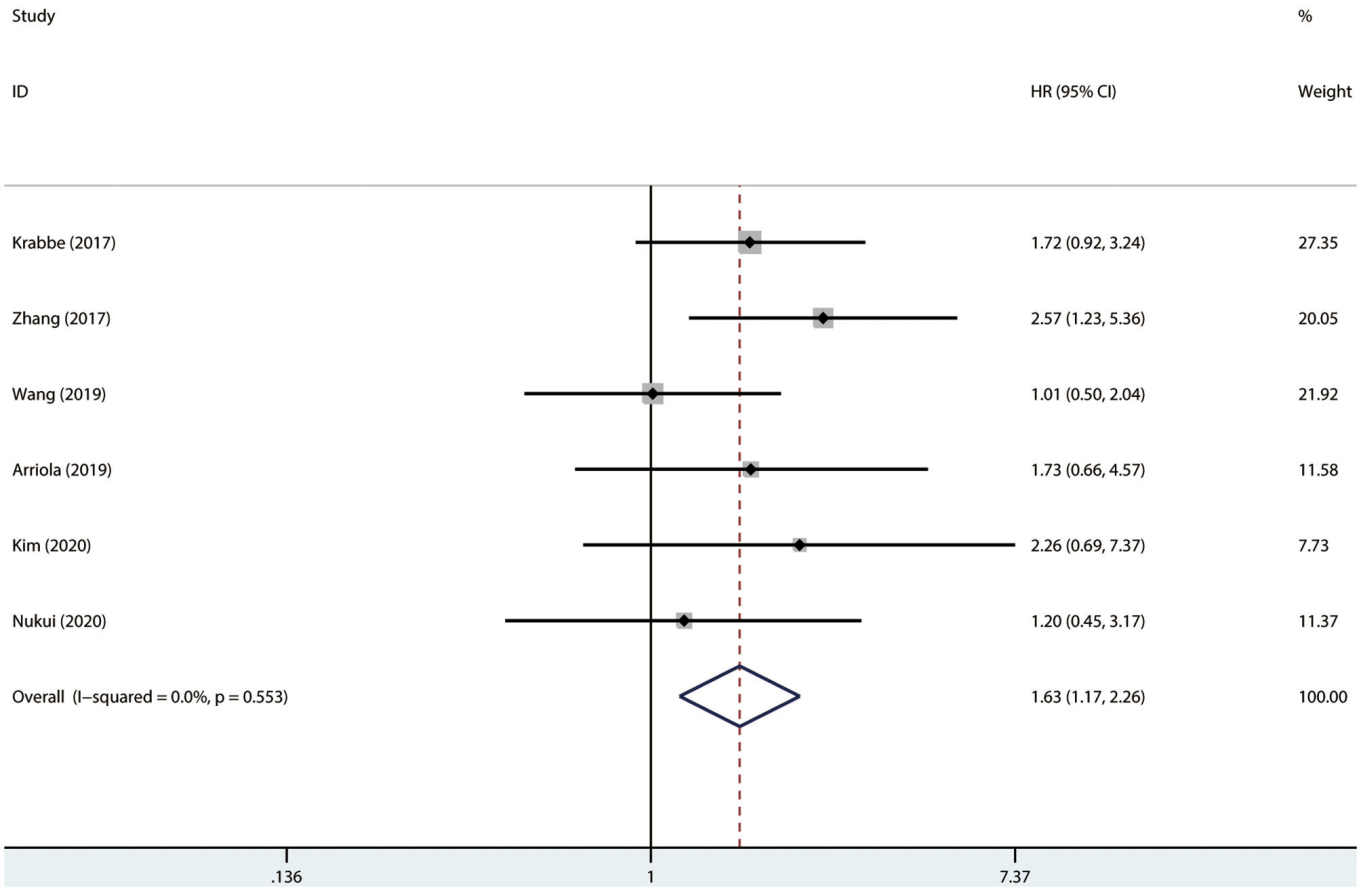
Prognostic value of PD-L1 for CSS. CSS, cancer-specific survival; HR, hazard ratio; CI, confidence interval.

Results were pooled and synthesized (summarized in Table 2). Six studies, with 1095 individuals, reported OS. We found that PD-L1 had no significant association with OS in radically resected UTUC patients (HR = 1.49, 95% CI = 0.76–2.91, *I*^2^ = 74.9%; Figure 3). Six studies, with 1135 individuals, reported CSS. The pooled results demonstrated that higher PD-L1 levels were related to shorter CSS (HR = 1.63, 95% CI = 1.17–2.26, *I*^2^ = 0.0%; Figure 4).

**Table 2:** Subgroup analyses between PD-L1 expression and survival outcomes.

	**OS**					**CSS**				
	**No. of studies**	**Pooled HR (95% CI)**	**Heterogeneity**		***P*-value for interaction**	**No. of studies**	**Pooled HR (95% CI)**	**Heterogeneity**		***P*-value for interaction**
			***I*^**2**^ (%)**	***P* value**				***I*^**2**^ (%)**	***P*-value**	
Overall	6	1.49 (0.76, 2.91)	74.9	0.001	–	6	1.63 (1.17, 2.26)	0.0	0.553	–
Year of publication					0.223					0.252
2018 and before	2	0.89 (0.50, 1.60)	58.3	0.122		2	2.04 (1.26, 3.29)	0.0	0.415	
After 2018	4	2.17 (0.71, 6.65)	73.8	0.010		4	1.33 (0.84, 2.09)	0.0	0.640	
Race					0.145					0.364
Caucasus	2	0.71 (0.49, 1.04)	0.0	0.694		2	1.72 (1.02, 2.92)	0.0	0.992	
Asian	4	2.11 (0.86, 5.17)	74.2	0.009		4	1.57 (1.03, 2.39)	23.1	0.273	
NOS score					0.301					0.237
8	3	1.21 (0.69, 2.15)	0.0	0.785		3	1.21 (0.74, 1.98)	0.0	0.678	
9	3	1.95 (0.56, 6.80)	89.5	0.000		3	2.07 (1.33, 3.22)	0.0	0.709	
Region					0.214					0.325
USA	2	0.71 (0.49, 1.04)	0.0	0.694		2	1.72 (1.02, 2.92)	0.0	0.992	
Japan	2	1.39 (0.78, 2.48)	0.0	0.674		1	1.20 (0.45, 3.18)	–	–	
China	1	1.11 (0.54, 2.28)	–	–		2	1.58 (0.95, 2.62)	69.2	0.072	
Korea	1	13.42 (3.64, 49.4)	–	–		1	2.26 (0.69, 7.39)	–	–	
Cutoff value					0.379					0.285
≥1%	3	0.78 (0.56, 1.09)	0.0	0.524		3	1.42 (0.93, 2.17)	0.0	0.492	
≥5%	3	2.85 (0.76, 10.68)	79.8	0.007		3	2.01 (1.19, 3.40)	0.0	0.462	

*OS, overall survival; CSS, cancer-specific survival*.

Results of meta-regression and subgroup analyses were presented in Table 2 and Supplementary Figures 1–8. No significant results were determined among the subgroups in terms of OS. The subgroup analysis by race indicated that higher PD-L1 (higher than cutoff values) was associated with shorter CSS in both Caucasians (HR = 1.72, 95% CI = 1.02–2.92, *I*^2^ = 0.0%) and Asians (HR = 1.57, 95% CI = 1.03–2.39, *I*^2^ = 23.1%). Moreover, results indicated that higher PD-L1 levels were associated with shorter CSS in the studies conducted in the USA (HR = 1.72, 95% CI = 1.02–2.92, *I*^2^ = 0.0%), but not in Japan (HR = 1.20, 95% CI = 0.45–3.18), China (HR = 1.58, 95% CI = 0.95–2.62, *I*^2^ = 69.2%), and Korea (HR = 2.26, 95% CI = 0.69–7.39). No significant difference was determined in any subgroup (*P*_interaction_ > 0.05 for all).

### PD-L1 and Tumor Behaviors of UTUC

Results on this were recorded in Table 3. We observed that PD-L1 had relationships with invasive depth (pT3+pT4+pT2 vs. pT1+pTa/pTis, OR = 2.53, 95% CI = 1.07–5.96, and *P* = 0.001) (Supplementary Figure 9) and tumor grade (High vs. Low, OR = 3.56, 95% CI = 1.82–6.97, *P* = 0.000; Supplementary Figure 10). However, PD-L1 levels had no significant associations with UTUC in terms of lymphovascular invasion (LVI; presence vs. absence, OR = 1.70, 95% CI = 0.77–3.78, and *P* = 0.132; Supplementary Figure 11), tumor location (pelvicalyceal vs. ureter, OR = 1.49, 95% CI = 0.99–2.26, and *P* = 0.102; Supplementary Figure 12), and gender (male vs. female, OR = 0.77, 95% CI = 0.55–1.09, and *P* = 0.122; Supplementary Figure 13). The pooled results on focality, concomitant CIS, recurrence, and metastasis were all not statistically associated with PD-L1 (*P* > 0.05 for all) (Supplementary Figures 14–17).

**Table 3:** Association between PD-L1 expression and clinicopathological features of UTUC.

**Items**	**No. of studies**	**Pooled OR (95% CI)**	***I*^**2**^ (%)**	***P-*value**	**Model**
Gender (male vs. female)	5	0.77 (0.55, 1.09)	0.0	0.586	Fixed
Depth of invasion (pT3+pT4+pT2 vs. pT1+pTa/pTis)	7	2.53 (1.07, 5.96)	84.9	0.000	Random
Grade (high vs. low)	4	3.56 (1.82, 6.97)	42.4	0.157	Fixed
Lymphovascular invasion (presence vs. absence)	5	1.70 (0.77, 3.78)	76.0	0.002	Random
Location (pelvicalyceal vs. ureter)	3	1.49 (0.99, 2.26)	0.0	0.871	Fixed
Focality (multifocal vs. unifocal)	2	0.96 (0.62, 1.49)	0.0	0.862	Fixed
Concomitant CIS (presence vs. absence)	2	1.28 (0.83, 1.97)	0.0	0.460	Fixed
Recurrence (presence vs. absence)	2	0.71 (0.46, 1.09)	0.0	0.886	Fixed
Tumor metastasis (presence vs. absence)	2	1.39 (0.68, 2.83)	0.0	0.428	Fixed

*OR, odds ratio; CI, confidence interval*.

### Publication Bias

**Figure 5:**
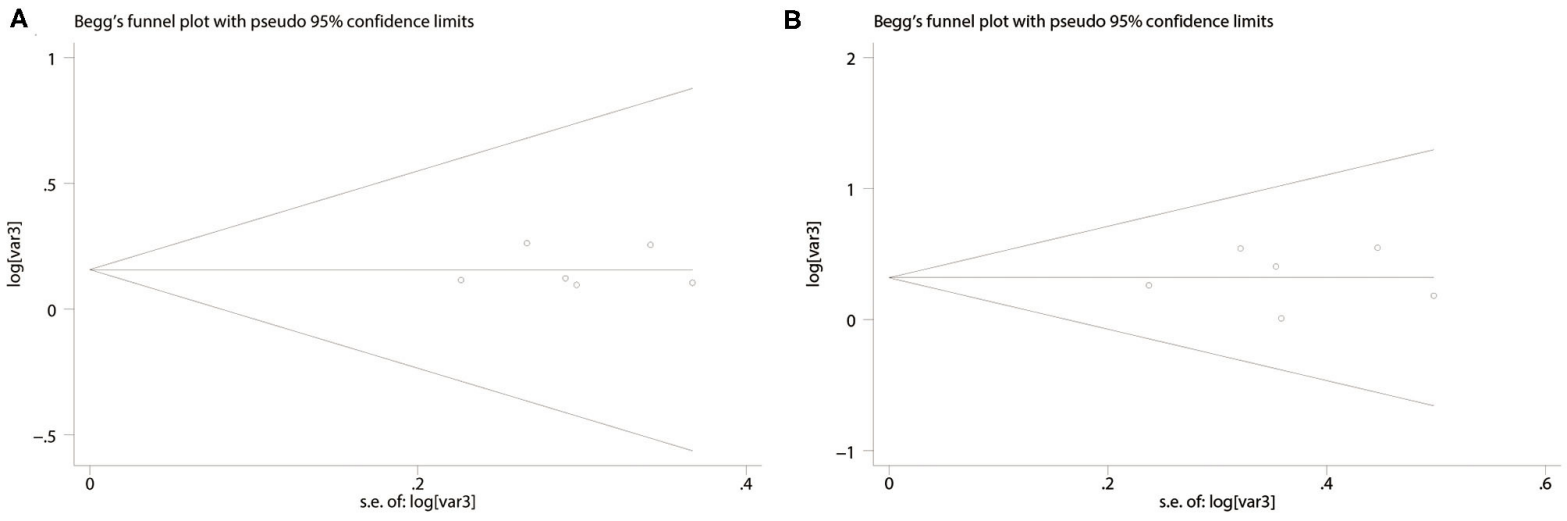
Funnel plots based on PD-L1 for overall survival **(A)** and cancer-specific survival **(B)**.

No significant publication bias was found [OS: Begg's test, *P* = 0.624; Egger's test, *P* = 0.558 (Figure 5A); CSS: Begg's test, *P* = 0.523; Egger's test, *P* = 0.425 (Figure 5B)].

### Sensitivity Analysis

**Figure 6:**
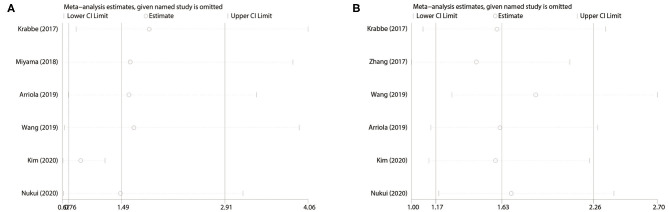
Sensitivity analysis. **(A)** PD-L1 for OS; **(B)** PD-L1 for CSS. OS, overall survival; CSS, cancer-specific survival.

**Figure 7:**
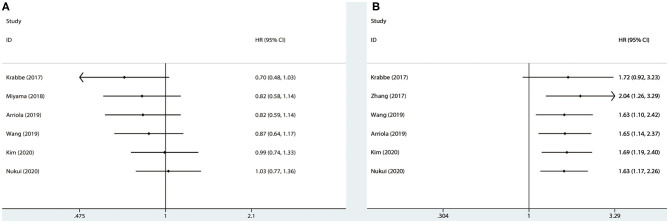
Cumulative meta-analysis for OS **(A)** and CSS **(B)**, based on year of publication. OS, overall survival; CSS, cancer-specific survival; HR, hazard ratio; CI, confidence interval.

We extracted each study subsequently in each analysis and found that any one study could not affect the pooled results; thus, the results were reliable (Figure 6). The cumulative meta-analysis was performed and was ordered by publication year (Figure 7). It revealed that higher PD-L1 levels were related to shorter CSS, not the OS. Furthermore, we also found that the 95% CIs narrowed as the pooled results gradually moved near the null.

## Discussion

To our knowledge, it is the first time to evaluate the relationship between PD-L1 and survival in radically resected UTUC patients through a meta-analysis. The pooled incidence of PD-L1 in UTUC patients was 21.0% (13.0–30.0%) and results indicated that higher PD-L1 was not related to OS but was associated with CSS for UTUC patients after nephroureterectomy. Notably, in the subgroup analysis, higher PD-L1 had associations with worse CSS in both Caucasian and Asian UTUC patients. Furthermore, higher PD-L1 had an association with both larger tumor and higher tumor grade of UTUC. In the meta-regression analysis, no related factors could significantly influence the pooled results. Results of the cumulative meta-analysis indicated that the 95% CIs narrowed as the pooled results gradually moved near the null.

In the meta-analysis, five studies reported a high expression rate (>20%) of PD-L1 (15, 17, 23–25), which provided rationality to apply immunotherapy in such cancers. Furthermore, the great variation in the prevalence might be ascribed to the different IHC strategies across studies, including the different cutoff values and diverse primary antibody species, etc. The small sample size of some included studies can also affect the pooled prevalence.

Up till now, studies reporting the role of PD-L1/PD-1 in the prognosis of UCs have focused mainly on urothelial carcinoma of the bladder (UCB) rather than UTUC (26). Moreover, these studies were often designed differently and evaluated different cells. Hayakawa et al. reported that positive PD-1 in the tumor nest was related to CSS and RFS (27). Two studies reported that PD-1 in tumor-infiltrating lymphocytes (TILs) and PD-L1 in tumor cells were linked with UTUC prognosis (15, 23). The meta-analysis only pooled the results from eight studies reporting PD-L1 levels from tumor cells and we found that higher PD-L1 levels had association with CSS rather than OS. Two previous large clinical trials showed in patients with advanced urothelial tumors that those who had higher PD-L1 had better objective response to pembrolizumab and nivolumab, which underlies the clinical implication for PD-L1 expression in UTUC (28, 29). To date, there were few studies in this area; more well-designed studies are warranted. In the subgroup analysis (both the OS and CSS) by race, both the studies with the Caucasian population (two studies in total) adopted ≥1% as a cutoff value, while three of the studies with Asian population (four studies in total) adopted ≥5% as a cutoff value and one used ≥1% as a cutoff value. Therefore, higher PD-L1 (≥1%) was related to shorter CSS in Caucasian UTUC cases after RNU. However, there were only few studies included in each subgroup. Notably, subgroup analysis by cutoff values was also performed (Table 2 and Supplementary Figures 18, 19). The pooled results indicated that positive PD-L1 had shorter CSS when the cutoff value was 5% (HR = 2.01, 95% CI = 1.19–3.40, *I*^2^ = 0.0%) and a trend between higher PD-L1 expression and shorter OS when studies used 1% as the cutoff (HR = 1.42, 95% CI = 0.93–2.17, *I*^2^ = 0.0%). However, considering that PD-L1 was tightly related to UCB survival and the similarity of UTUC with UCB (the same histology, etc.) (30), we tended to suppose that higher PD-L1 levels were related to worse prognosis in the UTUC population.

Currently, checkpoint inhibitors have been widely rationalized in several cancers partly because of its well objective response rates and favorable efficacy (31). If PD-L1 levels in tumor cells were linked to clinical and pathological features, the PD-L1 inhibitors would inhibit the tumor biology, such as invasion, recurrence, and metastasis, etc. In the study, PD-L1 was related to UTUC in T stage and tumor grade, and similar findings have been indicated in both UTUC and other types of tumors (23, 24, 32, 33). Results indicated that PD-L1 could not promote the LVI of UTUC, while great heterogeneity existed in the five studies (13, 15–17, 24). However, previous studies have demonstrated that high PD-L1 was related to presence of LVI of tumors (15, 34, 35). Furthermore, we found that UTUC with higher PD-L1 was more likely to present at pelvicalyceal. The results may provide evidence for immunotherapy and rationalizing it as a promising perioperative therapy for UTUC. However, due to the small number of studies (≤3) investigating these results, the real associations are still elusive and more studies are required.

Factors, such as non-surgical treatments, Chinese herb exposure, etc. in original studies may potentially affect the pooled results. Some differences in the treatments for UTUC existed among the eight included studies. Few of them specify the protocols of the therapies and whether the patients had received neoadjuvant chemotherapy (NC) or adjuvant chemotherapy (AC) or immunotherapy. Only one study clearly demonstrated that their patients only received surgical treatment and made a discussion on this through comparisons with similar researches (15). The study conducted by Skala et al. incorporated AC and NC into a multivariate Cox regression model, which reduced the influence on HR, while the immunotherapy was not clearly reported (24). Detailed specification on the treatment information was important to better evaluate the association between immune biomarkers and prognosis, because all these non-surgical therapies may potentially influence the survival outcomes and NC may affect the PD-L1 levels or tumor biology. Based on the variations, it might be difficult to evaluate the impact of different treatments on the pooled results by conducting subgroup analysis. The concerns should be considered and future well-designed studies that specified the treatments are expected to investigate the issue better.

Our findings have some research and clinical implications. Firstly, expression of PD-L1 may be a meaningful marker for prognosis in UTUC cases after radical resection. We can utilize PD-L1 to predict survival outcomes, patients with positive PD-L1 may tend to show more advanced tumor features and a potentially worse prognosis. Secondly, PD-L1 expression may not be the only biomarker associated with prognosis in the population. Future studies need to concentrate on the PD-1/PD-L1 pathway or other molecules, instead of the single protein. Thirdly, PD-1/PD-L1 blockades could be an effective treatment selection for UTUC patients with positive PD-L1 after RNU, especially in the era of defining efficient postoperative treatments. Currently, PD-1/PD-L1 blockades have been considered as the replacement of conventional chemotherapy in several cancers (36). As early as 2012, Birtle et al. (37) started to make contributions in the postoperative care for UTUC by introducing the POUT trial, and aimed to define a standard treatment and improve the survival. In April 2020, recent data from the POUT trial, which explored the efficacy of platinum-based chemotherapy as a post-operative therapy for radically resected UTUC, indicated that gemcitabine–platinum combination chemotherapy initiated within 3 months could significantly improve survival with acceptable adverse effects and without dramatic transient in life quality in locally advanced UTUC patients after the primary surgery, which represented that the chemotherapy can be applied to clinical practice as a novel standard of treatment for UTUC patients after the RNU (38). The meta-analysis with the same population as the POUT trial urged the need for prospective studies or clinical trials assessing the association between PD-L1 and survival or related immunotherapy in the population. Additionally, to select appropriate therapies, we should also take clinicopathological features, individual preferences, treatment and related histories, and adverse effects into consideration.

Our study has some strengths. Firstly, the reliability of the results was repeatedly validated in the methodology. Secondly, all included studies measured PD-L1 levels by IHC. Of note, IHC is the major and the most widely applied method to evaluate the expression of protein. Therefore, the findings are clinically performable.

Finally, limitations should be mentioned. First and foremost, heterogeneity originating from the different cutoff values and sometimes the differences in primary antibody species, makes it difficult to reach a solid conclusion; 10, 5, and 1% were often used as the cutoff value of positive PD-L1. Three eligible methods could be applied to minimize the errors: (a) setting a unified standard on the cutoff value; (b) selecting a single cutoff value in one meta-analysis at a time; (c) conducting the subgroup analysis by cutoff value. Secondly, the study focused on PD-L1 from tumor cells. Therefore, PD-L1 or PD-1 expressed by other cells or tissues in tumor remains to be explored. Thirdly, the great inconsistency in treatments may potentially influence the survival outcomes and PD-L1 levels, as well as tumor biology, although with the above limitations, the publication bias and sensitivity analysis indicated the reliability of the results.

In conclusion, positive rate of PD-L1 21.0% (95% CI: 13.0–30.0%) in UTUC. Higher PD-L1 levels in tumor cells were related to shorter CSS in UTUC patients and the invasive depth (T stage) and tumor grade of UTUC. Incorporating PD-L1 into prognostic tools might improve the survival of UTUC by helping to select appropriate adjuvant treatments. The study reported the prognostic significance of PD-L1 in UTUC patients who received RNU. However, given the limitations in the meta-analysis, more studies investigating the relationship between PD-1/PD-L1 and prognosis in the population are expected in the promising field.

## Data Availability Statement

The original contributions presented in the study are included in the article/Supplementary Material, further inquiries can be directed to the corresponding author/s.

## Author Contributions

Conception: YL and ZLu. Administrative support: XL. Collection: YS, YL, JT, JK, and ZLi. Data analysis and interpretation: LL, YS, XW, and YY. Manuscript writing, revision, and approval: All authors.

## Conflict of Interest

The authors declare that the research was conducted in the absence of any commercial or financial relationships that could be construed as a potential conflict of interest.
