# Supplementary material for: The Prevalence and Prognostic Role of PD-L1 in Upper Tract Urothelial Carcinoma Patients Underwent Radical Nephroureterectomy: A Systematic Review and Meta-Analysis
Source: Front Oncol. 2020 Aug 21;10:1400. doi: 10.3389/fonc.2020.01400 (PMC7472102; doi:10.3389/fonc.2020.01400)
Supplement: Supplementary file 1 [file Data_Sheet_1.docx]

Supplementary Material

## Supplementary Tables

**Supplementary Table 1 Systematic search strategy (PICOS strategy).**

| Search strategy |  |
| --- | --- |
| Population | #1 ((Upper tract OR Upper urinary tract OR Renal pelvis OR Ureter) AND (Urothelial carcinoma OR Transitional cell carcinoma OR Carcinoma OR Cancer) OR "Carcinoma, Transitional Cell"[Mesh])) |
| Intervention | #2 CD274 OR programmed cell death ligand 1 OR B7-H1 OR PD-L1 OR B7 homolog 1 |
| Comparison | High vs. low expression of PD-L1 in tumor tissue |
| Outcomes | #3 Survival OR prognostic OR prognosis OR outcome |
| Study design | Randomized controlled trials, controlled clinical trials, prospective and retrospective cohort studies |
| Search combination | #1 AND #2 AND #3 |

**Supplementary Table 2 The Newcastle-Ottawa scale (NOS) quality assessment of the included studies.**

| Study (first autor,year) | Study design | Selection | Comparability | Outcomes | Total |
| --- | --- | --- | --- | --- | --- |
| Skala 2016 | RC | 4 | 2 | 2 | 8 |
| Krabbe 2017 | RC | 4 | 2 | 3 | 9 |
| Zhang 2017 | RC | 4 | 2 | 3 | 9 |
| Miyama 2018 | RC | 4 | 2 | 3 | 9 |
| Arriola 2019 | RC | 3 | 2 | 3 | 8 |
| Wang 2019 | RC | 3 | 2 | 3 | 8 |
| Nukui 2020 | RC | 3 | 2 | 3 | 8 |
| Kim 2020 | RC | 4 | 2 | 3 | 9 |

RC: retrospective cohort.

## Supplementary Figures

##
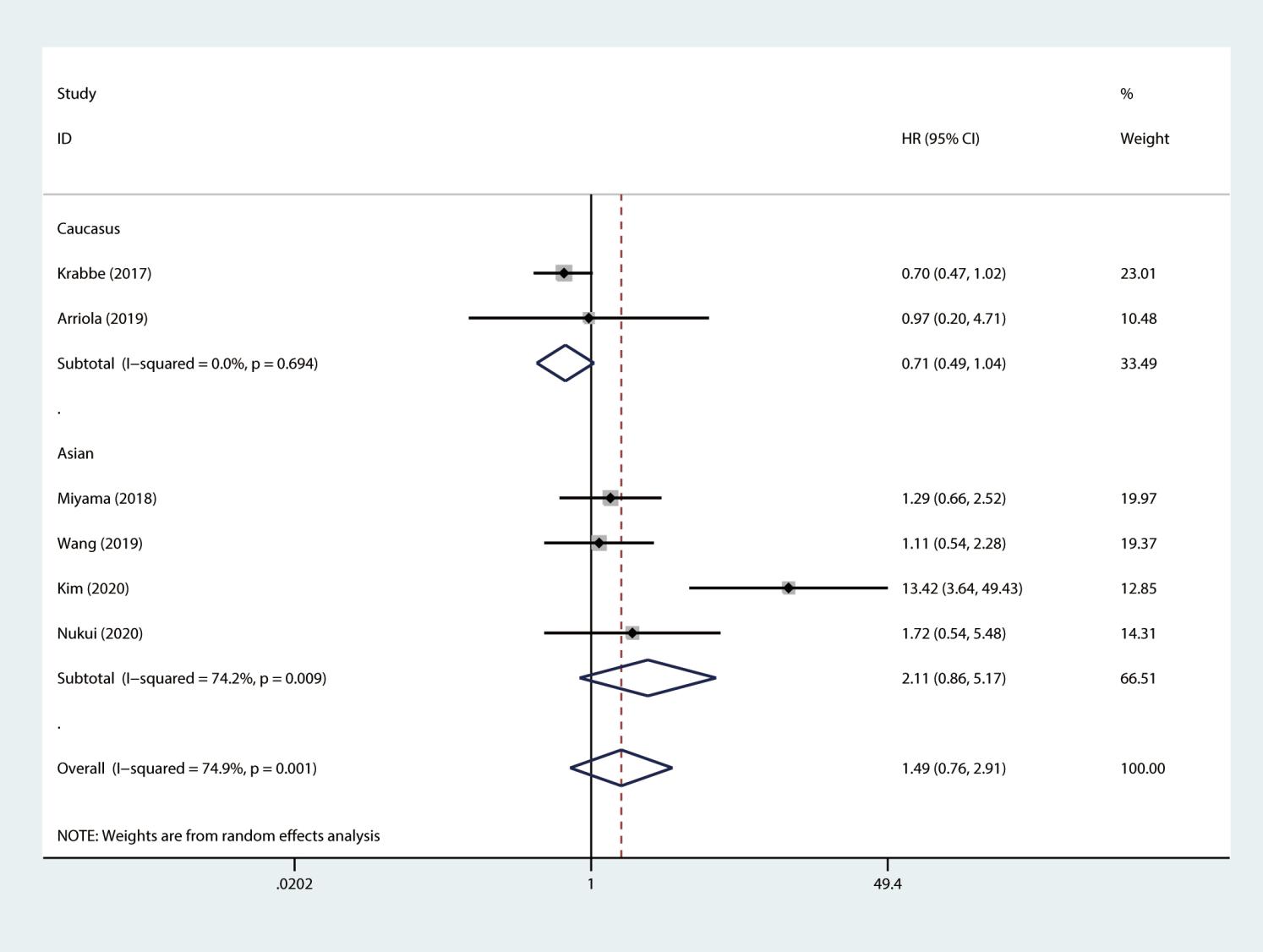


## Supplementary Figure 1. Subgroup analysis between PD-L1 expression and OS by race.

HR: hazard ratio; CI: confidence interval.


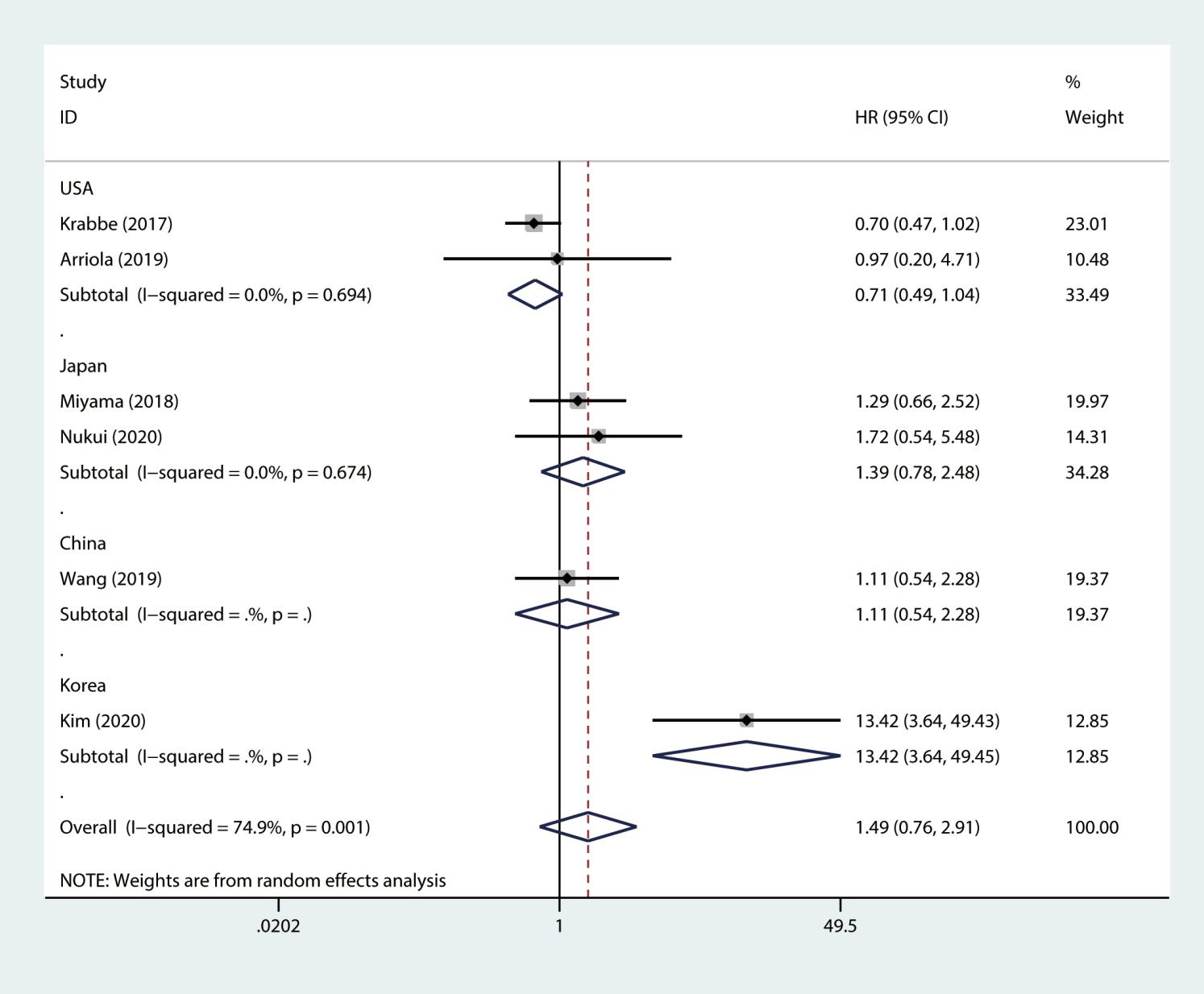


## Supplementary Figure 2. Subgroup analysis between PD-L1 expression and OS by study region.

HR: hazard ratio; CI: confidence interval.


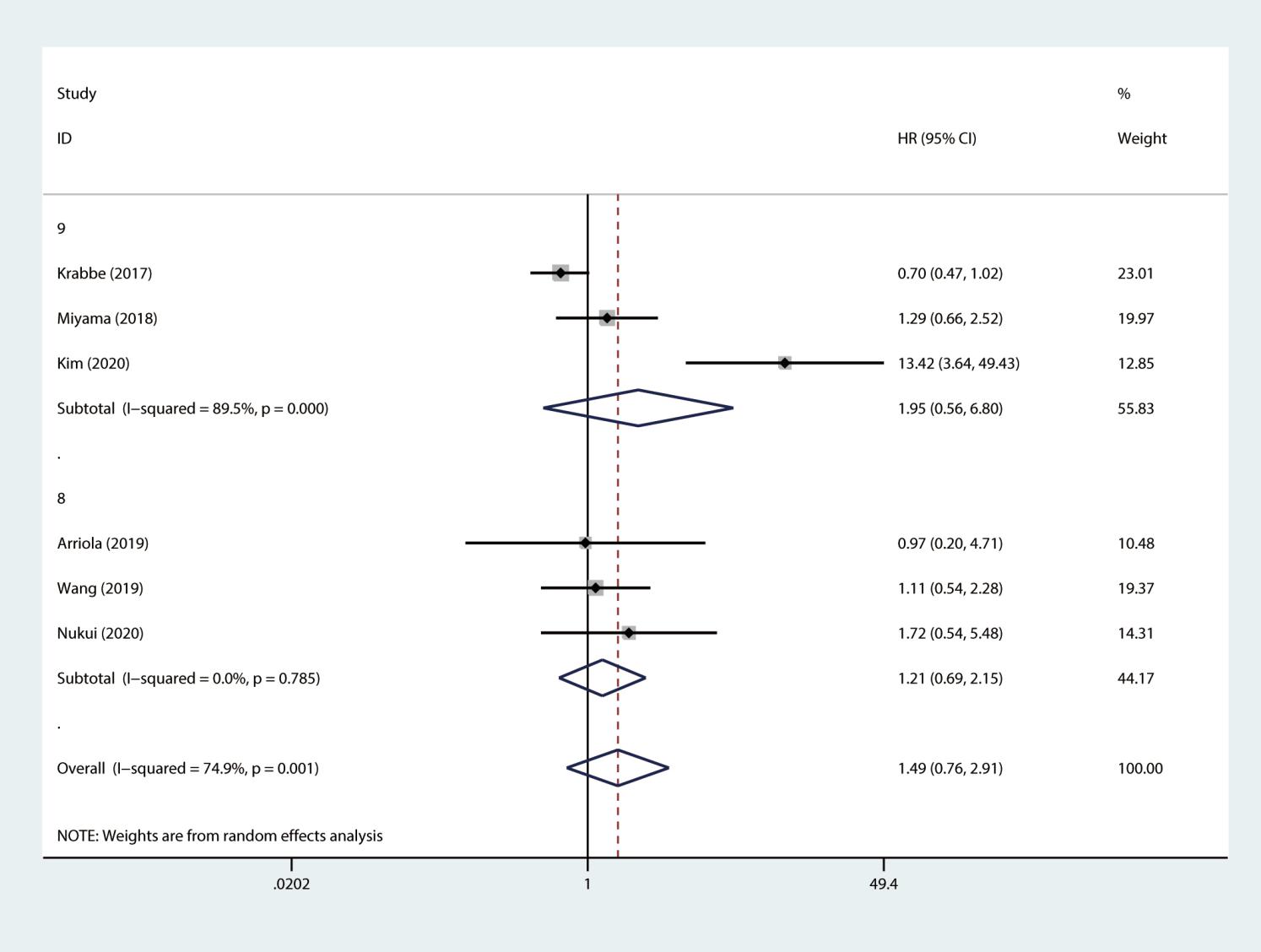


## Supplementary Figure 3. Subgroup analysis between PD-L1 expression and OS by NOS grades.

HR: hazard ratio; CI: confidence interval.


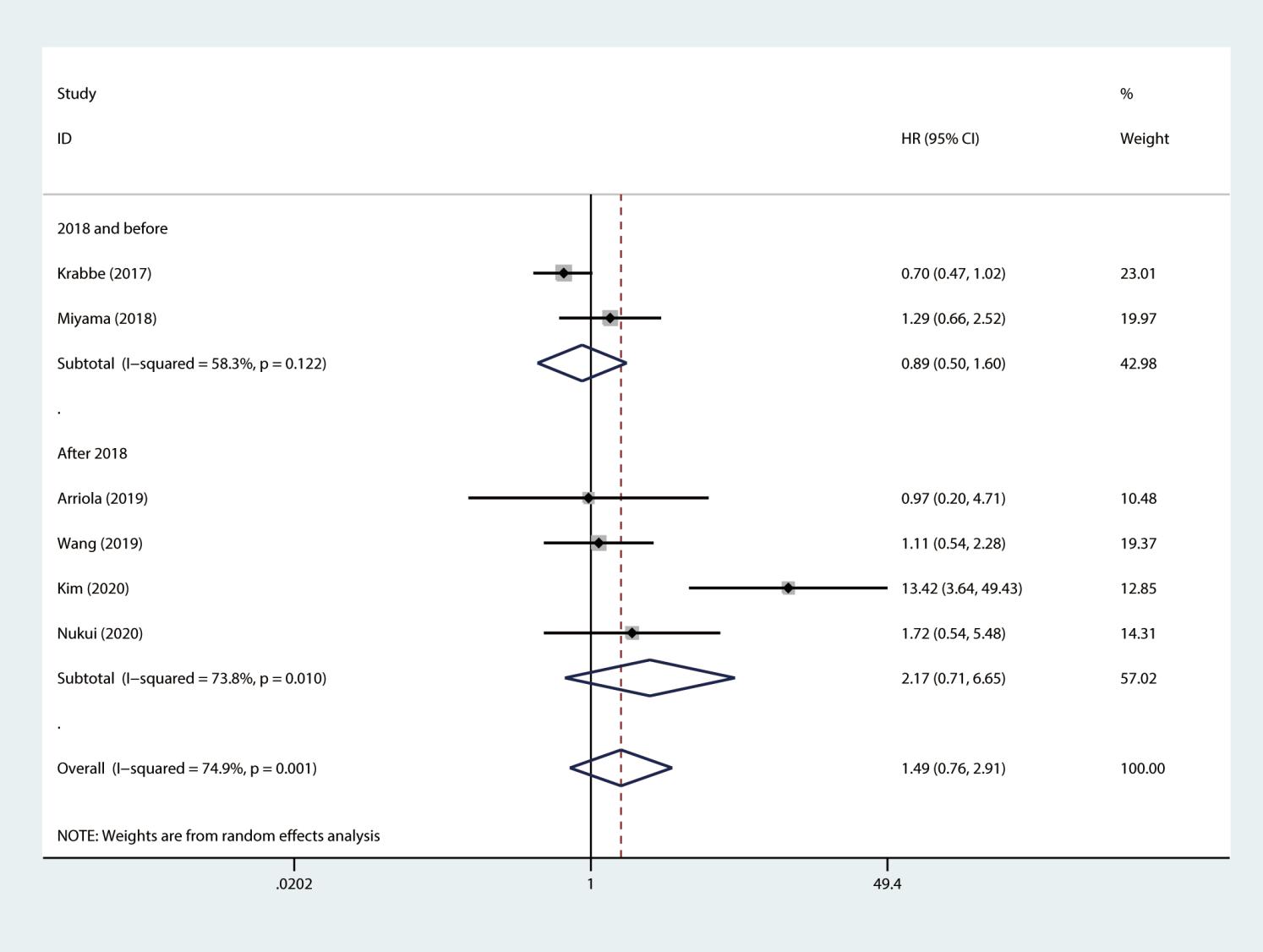


## Supplementary Figure 4. Subgroup analysis between PD-L1 expression and OS by publication years.

HR: hazard ratio; CI: confidence interval.


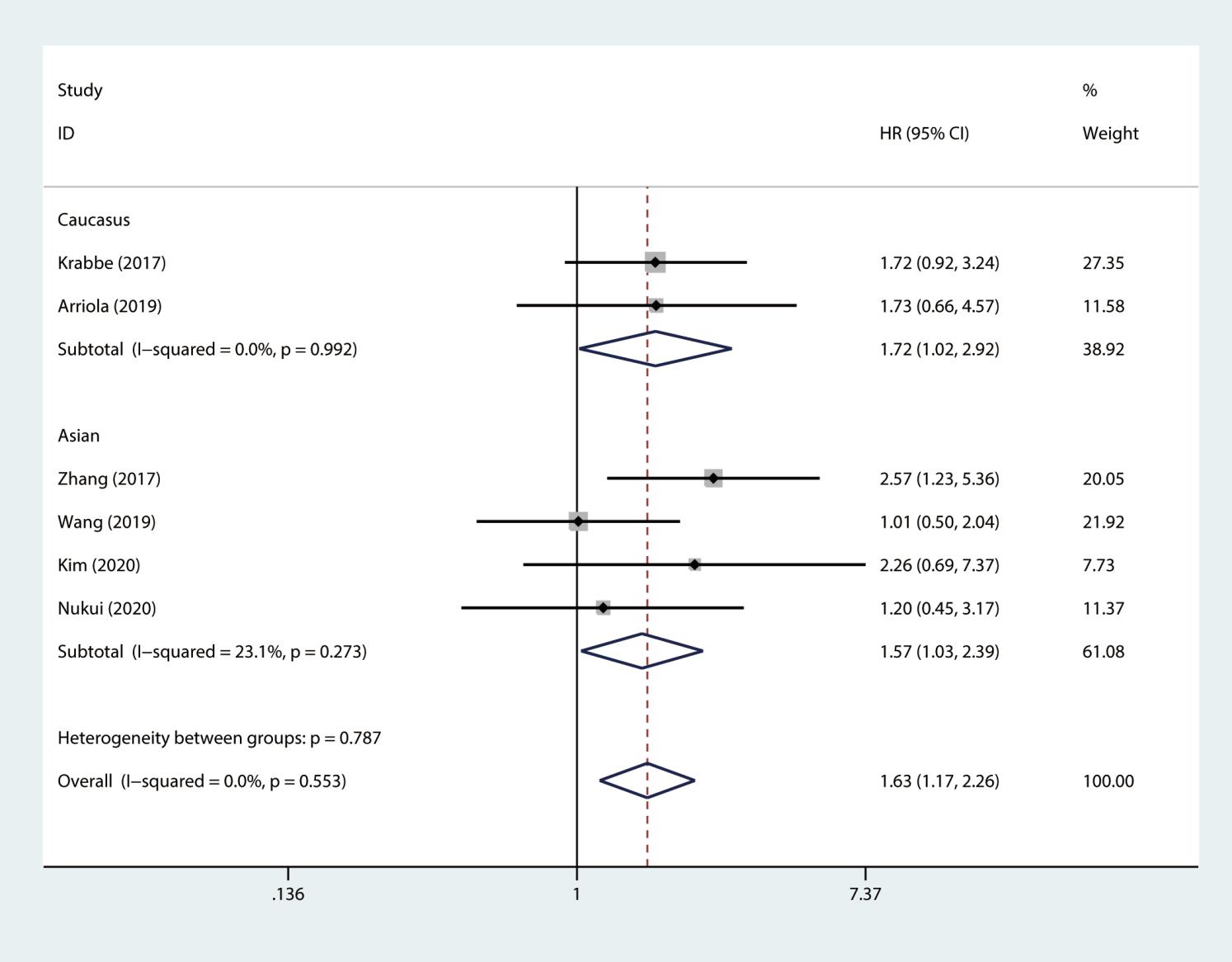


## Supplementary Figure 5. Subgroup analysis between PD-L1 expression and CSS by race.

HR: hazard ratio; CI: confidence interval.


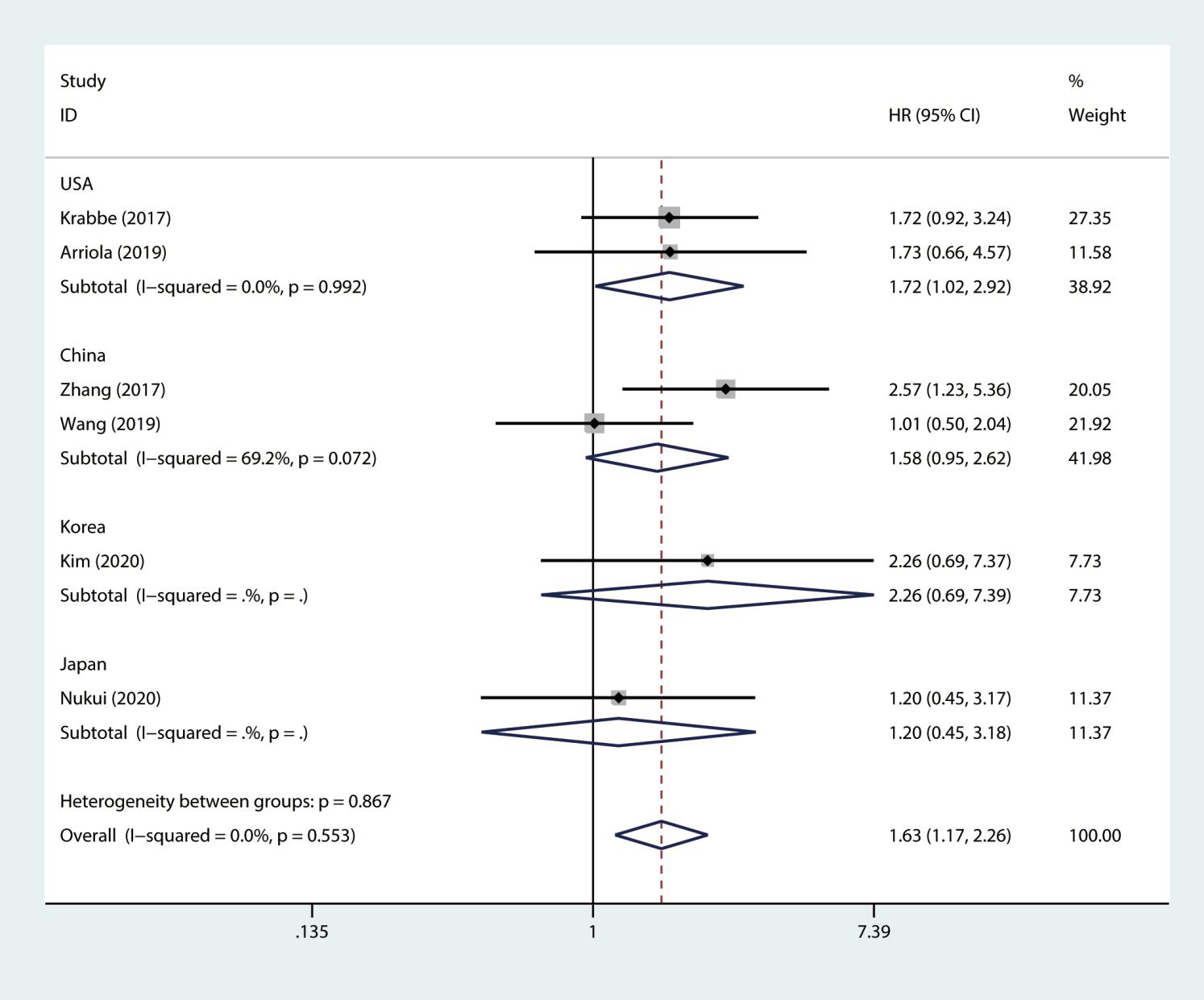


## Supplementary Figure 6. Subgroup analysis between PD-L1 expression and CSS by study region.

HR: hazard ratio; CI: confidence interval.


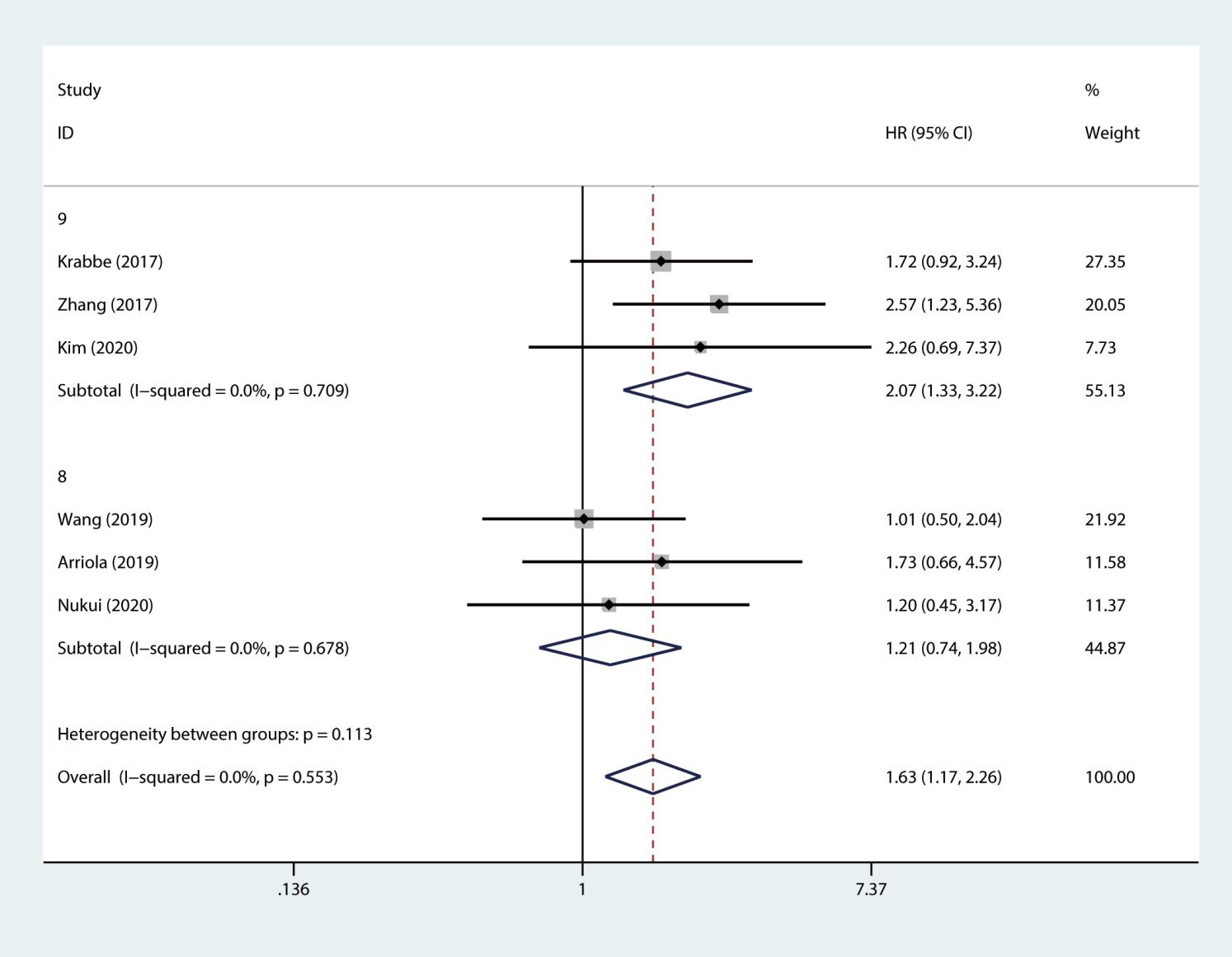


## Supplementary Figure 7. Subgroup analysis between PD-L1 expression and CSS by NOS grades.

HR: hazard ratio; CI: confidence interval.


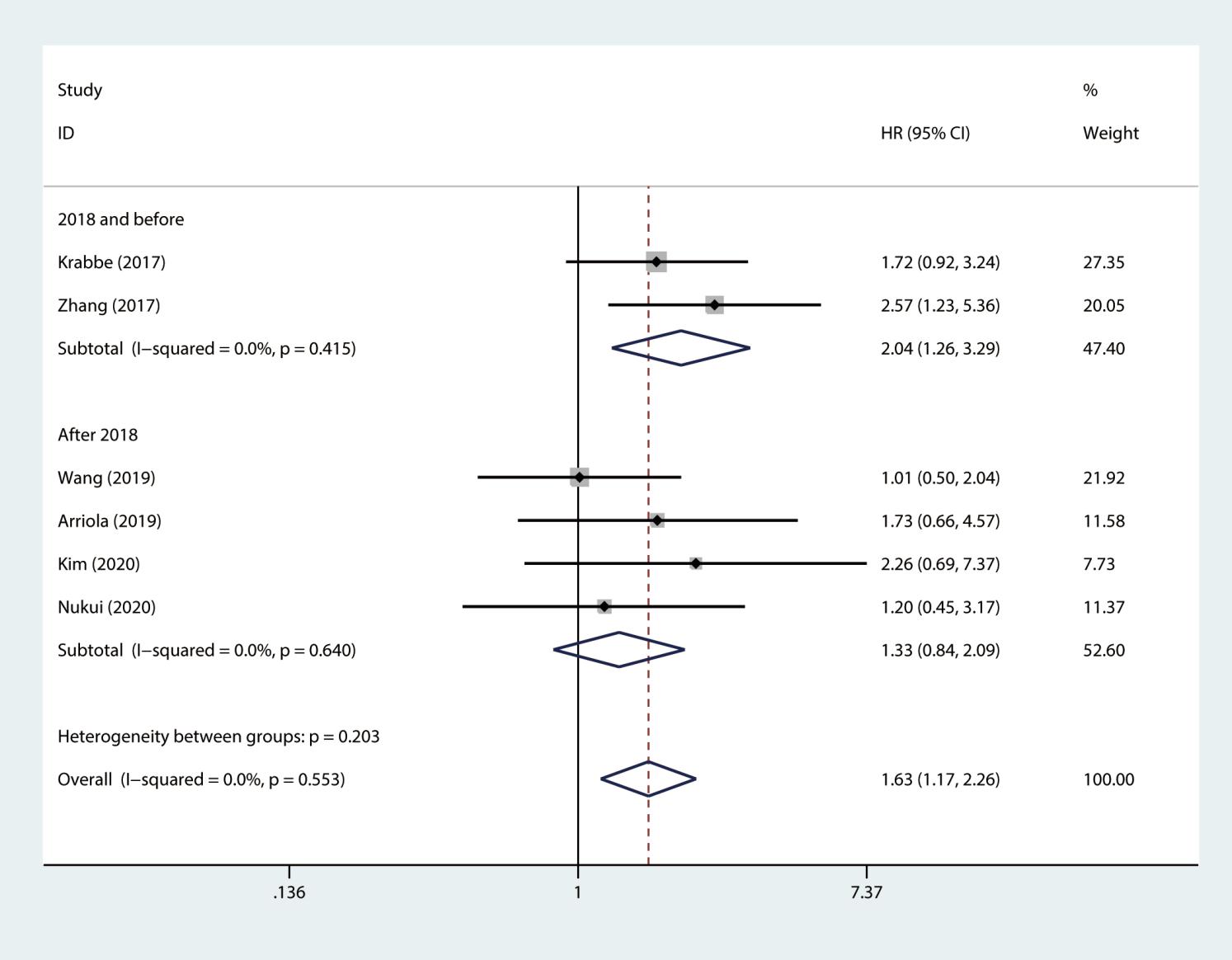


## Supplementary Figure 8. Subgroup analysis between PD-L1 expression and CSS by publication years.

HR: hazard ratio; CI: confidence interval.


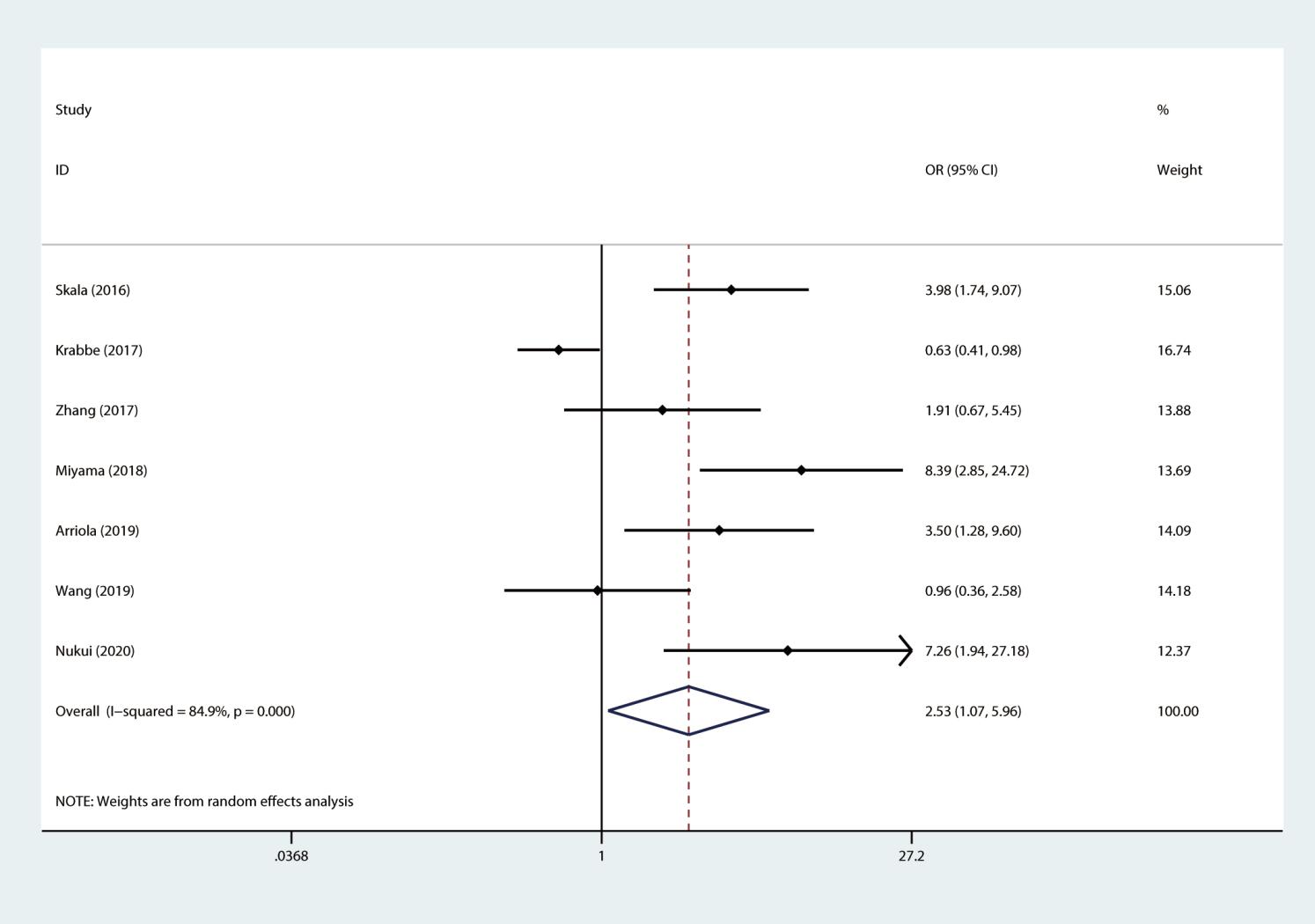


## Supplementary Figure 9. Relationship between PD-L1 expression and T stage of UTUC.

OR: odds ratio; CI: confidence interval.


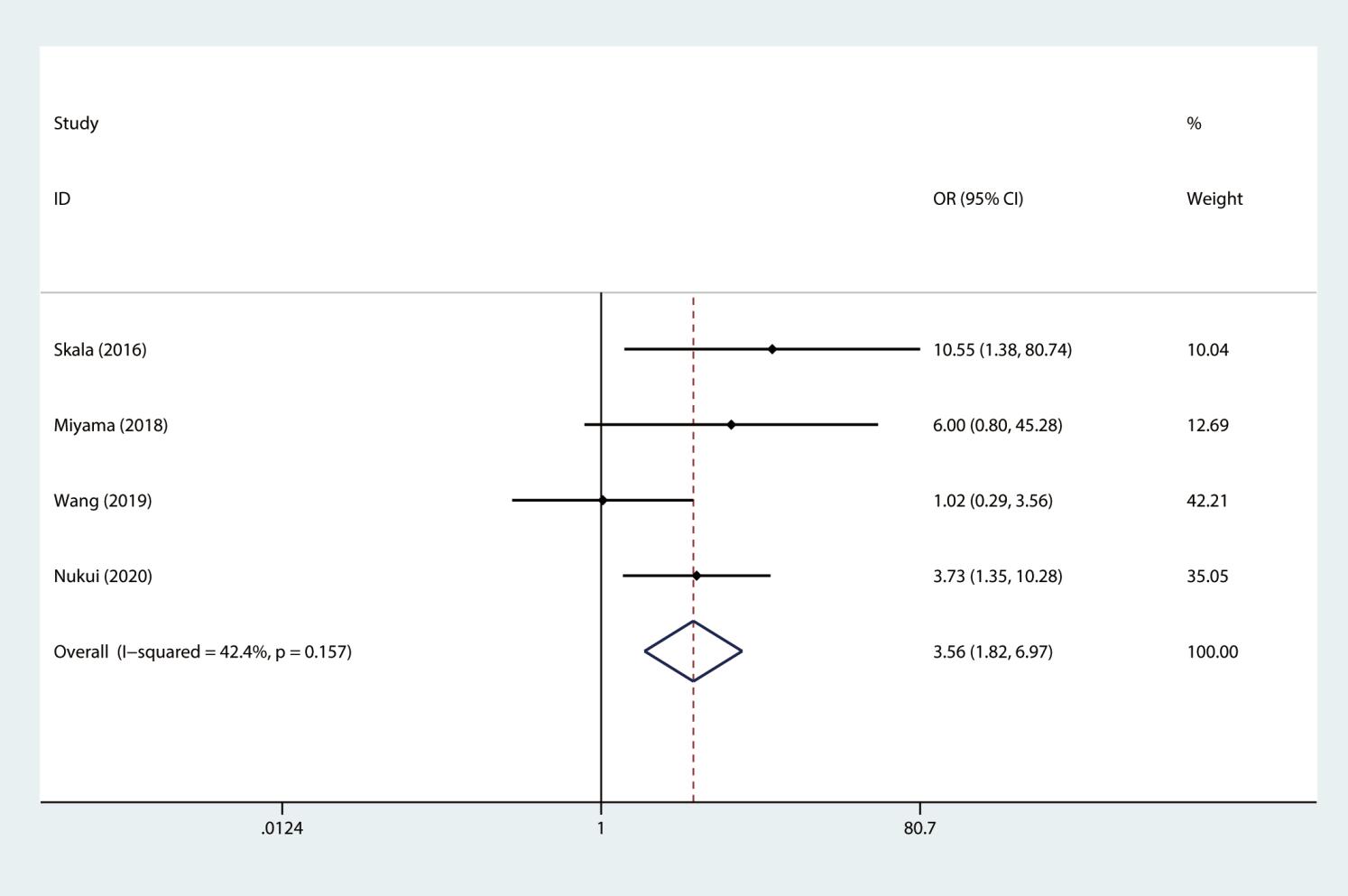


## Supplementary Figure 10. Relationship between PD-L1 expression and the grade of UTUC.

OR: odds ratio; CI: confidence interval.


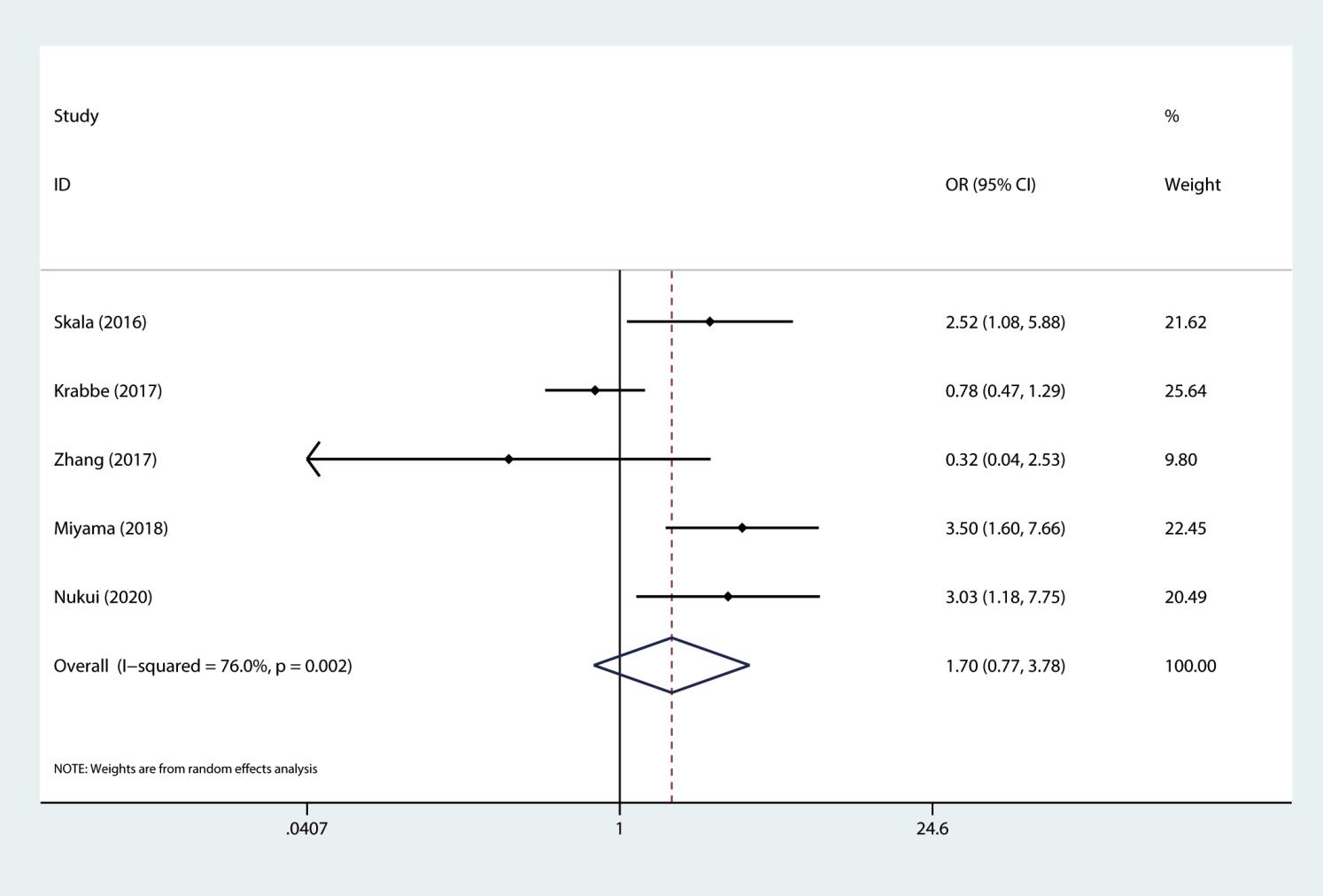


## Supplementary Figure 11. Relationship between PD-L1 expression and lymphovascular invasion (LVI) of UTUC.

OR: odds ratio; CI: confidence interval.


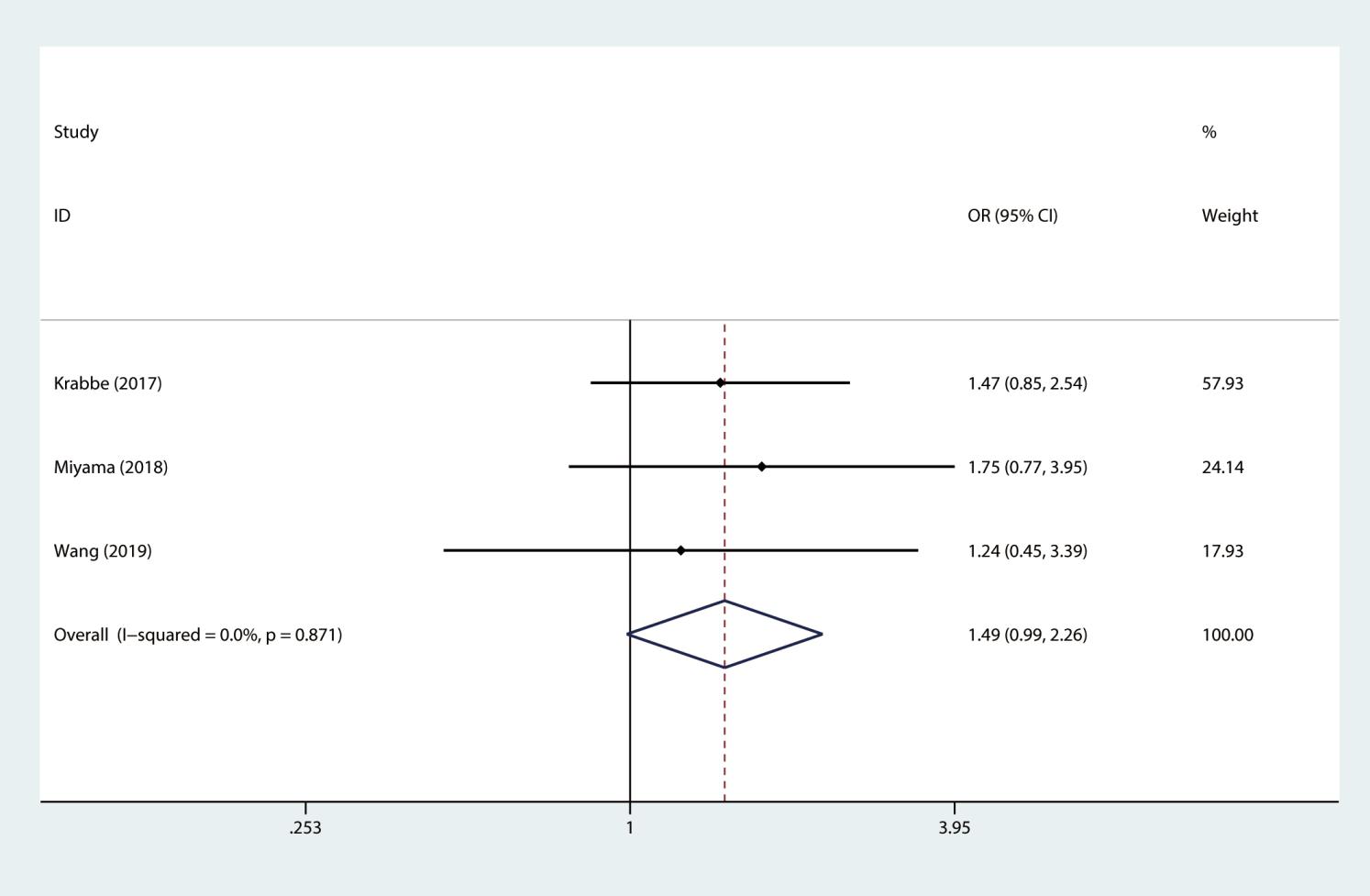


## Supplementary Figure 12. Relationship between PD-L1 expression and the primary location of UTUC.

OR: odds ratio; CI: confidence interval.


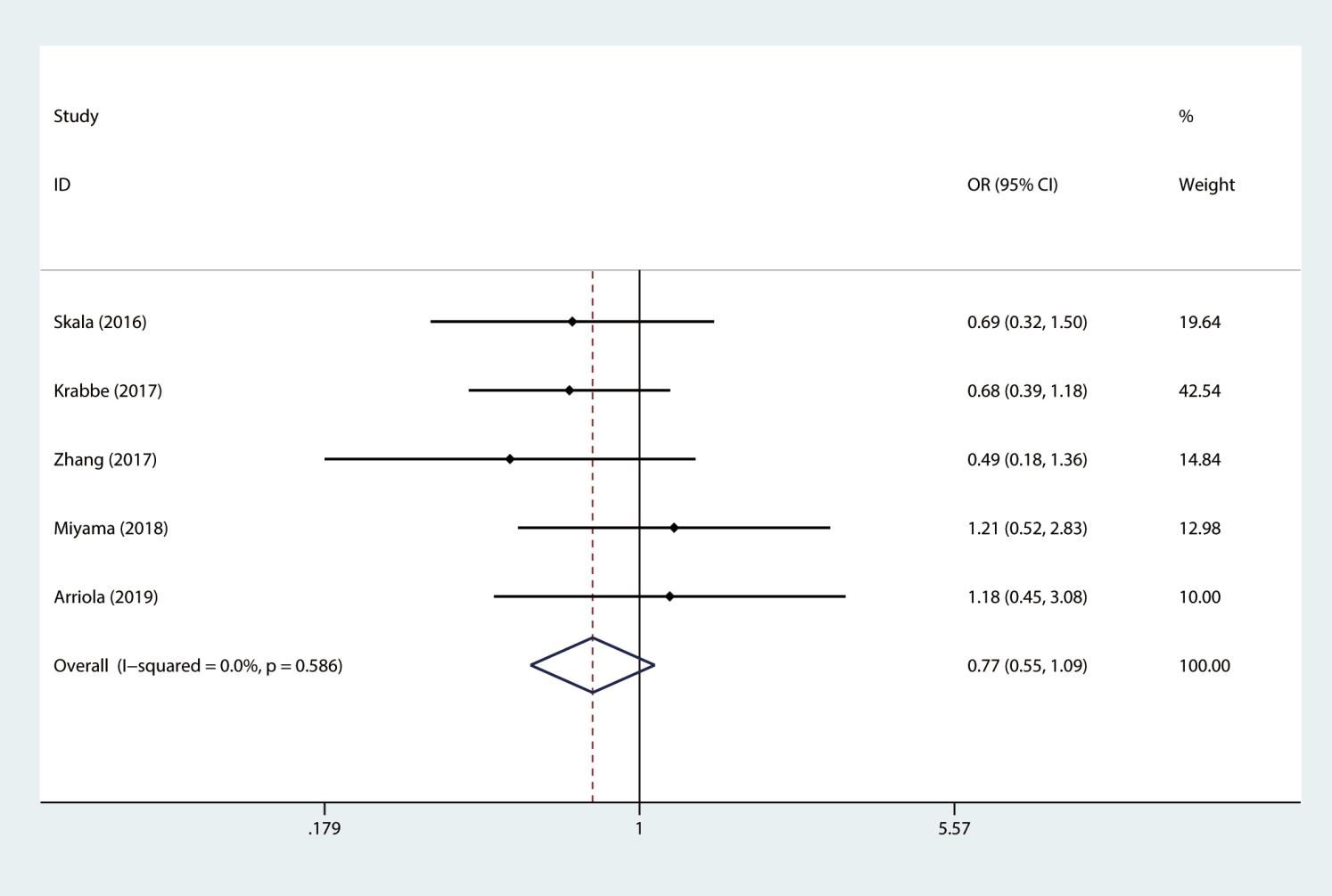


## Supplementary Figure 13. Relationship between PD-L1 expression and gender of UTUC patients.

OR: odds ratio; CI: confidence interval.


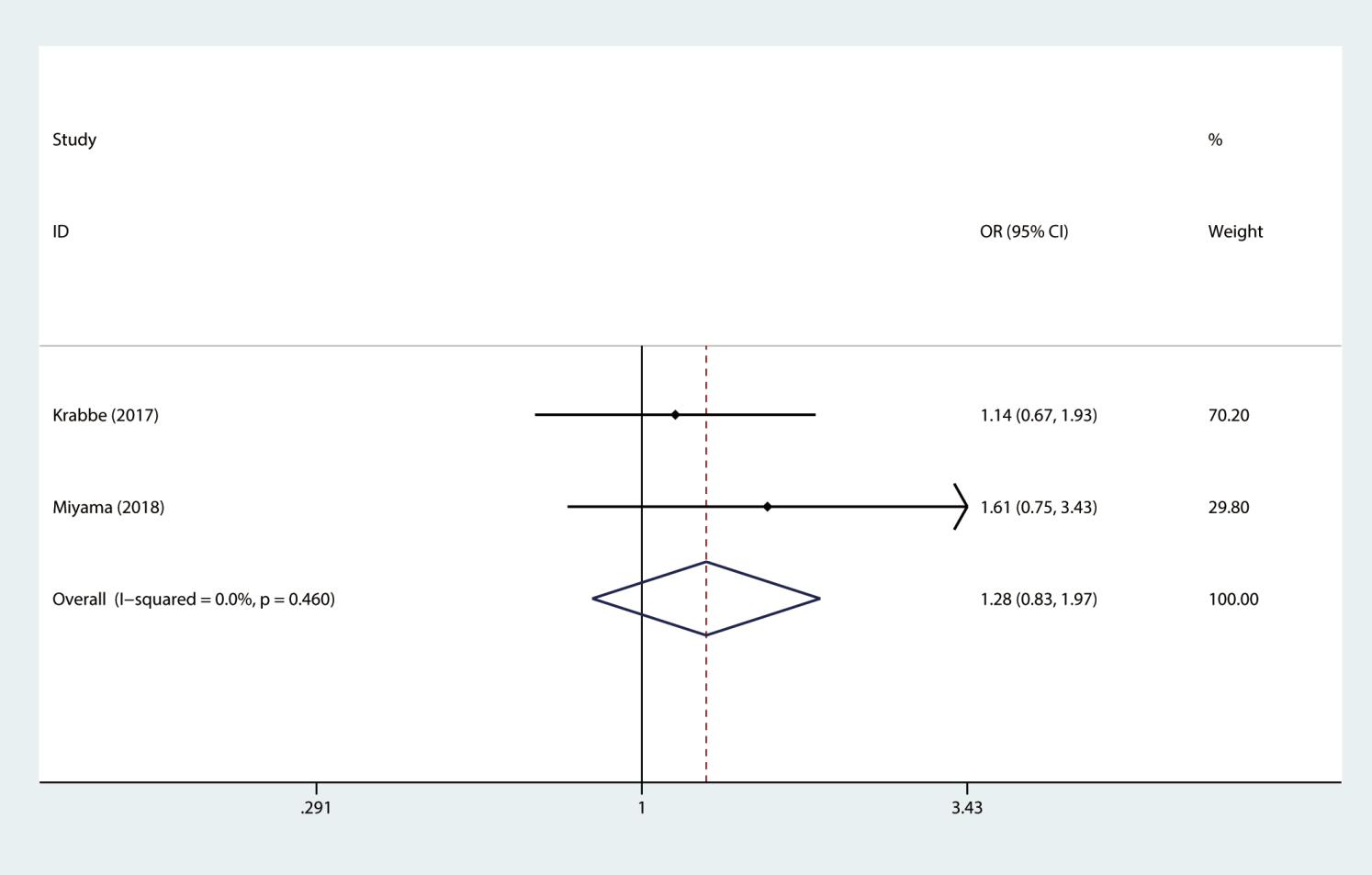


## Supplementary Figure 14. Relationship between PD-L1 expression and concomitant CIS of UTUC.

OR: odds ratio; CI: confidence interval; CIS: carcinoma in situ.


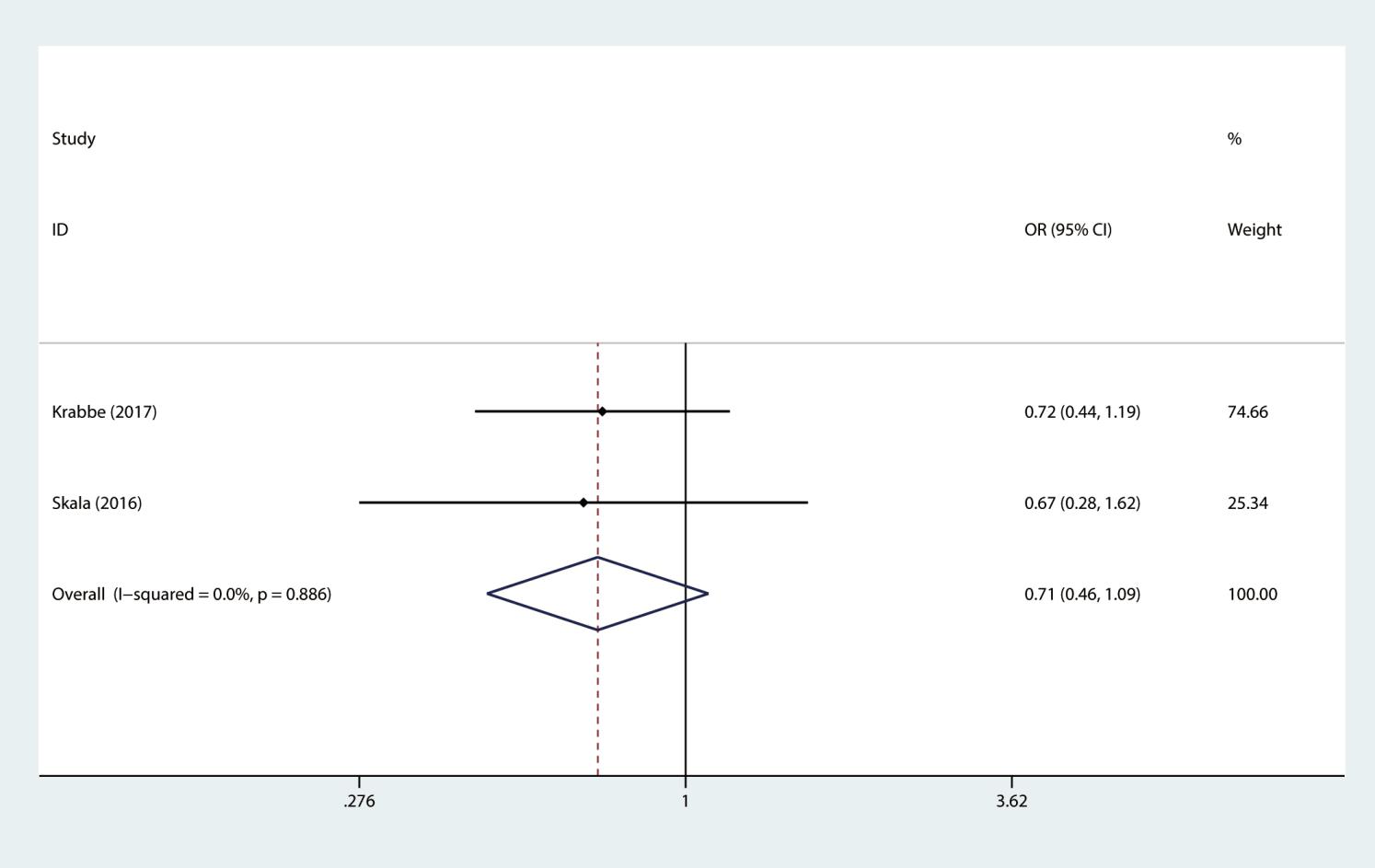


## Supplementary Figure 15. Relationship between PD-L1 expression and the recurrence of UTUC.

OR: odds ratio; CI: confidence interval.


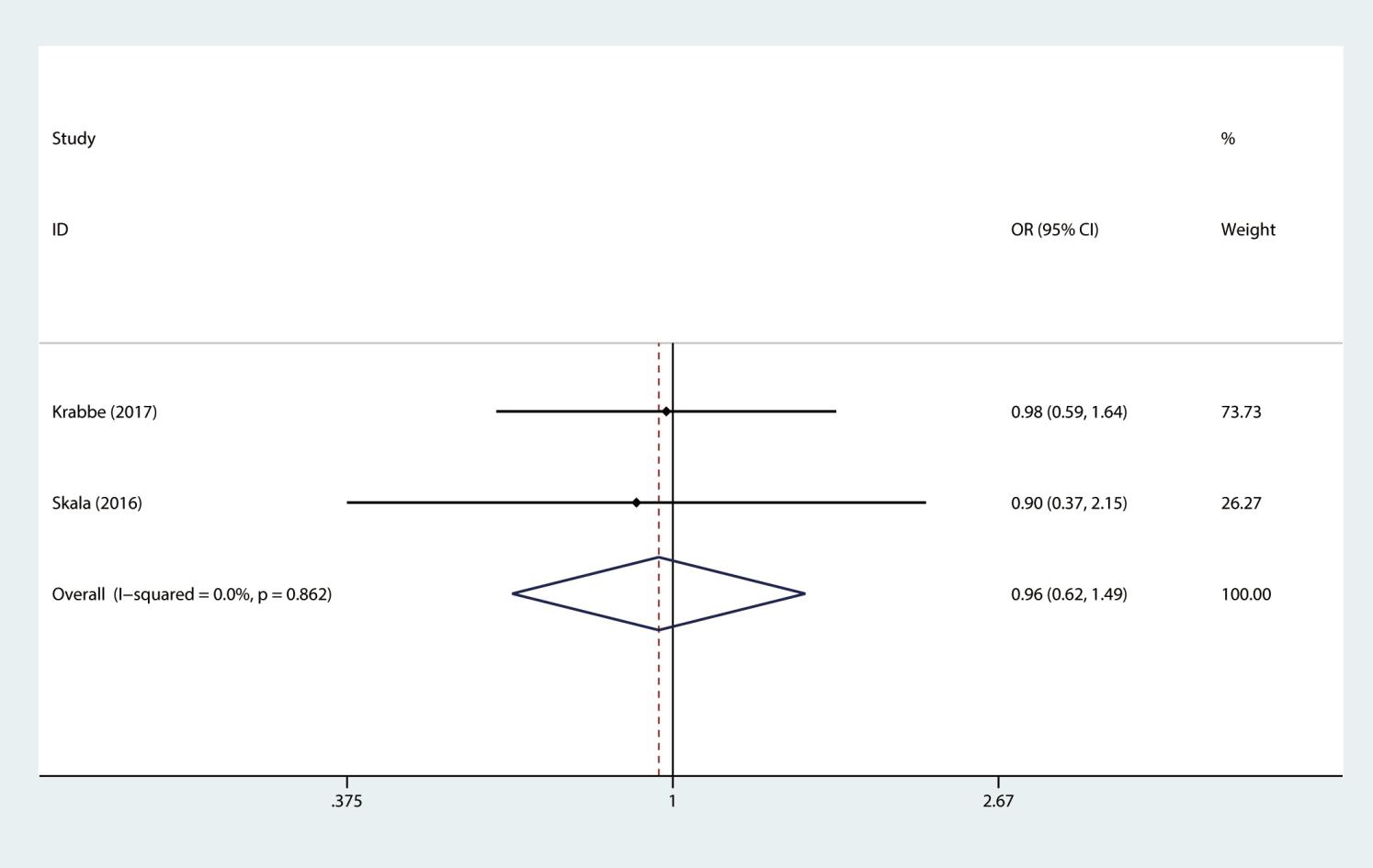


## Supplementary Figure 16. Relationship between PD-L1 expression and the focality of UTUC.

OR: odds ratio; CI: confidence interval.


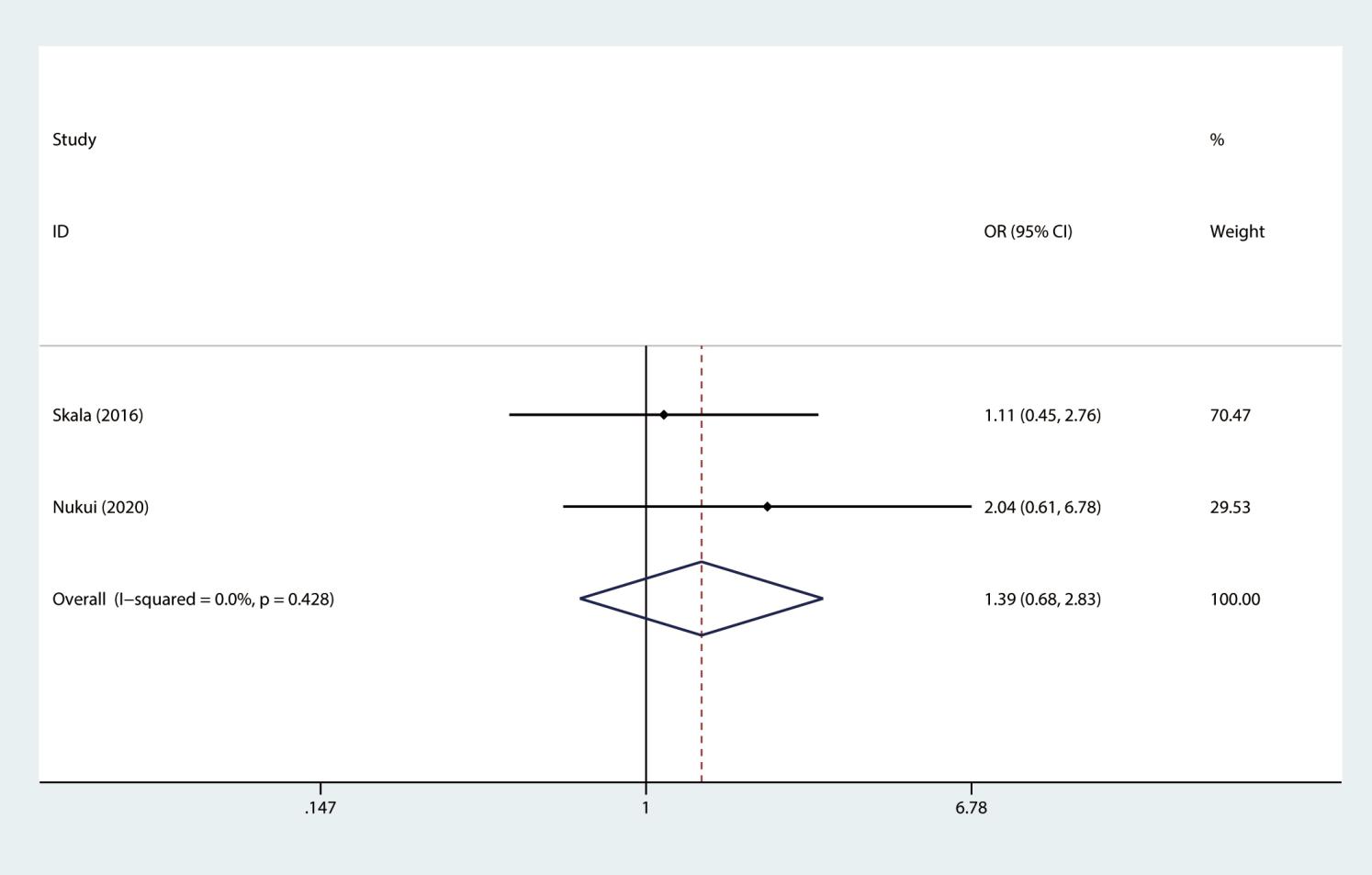


## Supplementary Figure 17. Relationship between PD-L1 expression and the metastasis of UTUC.

OR: odds ratio; CI: confidence interval.


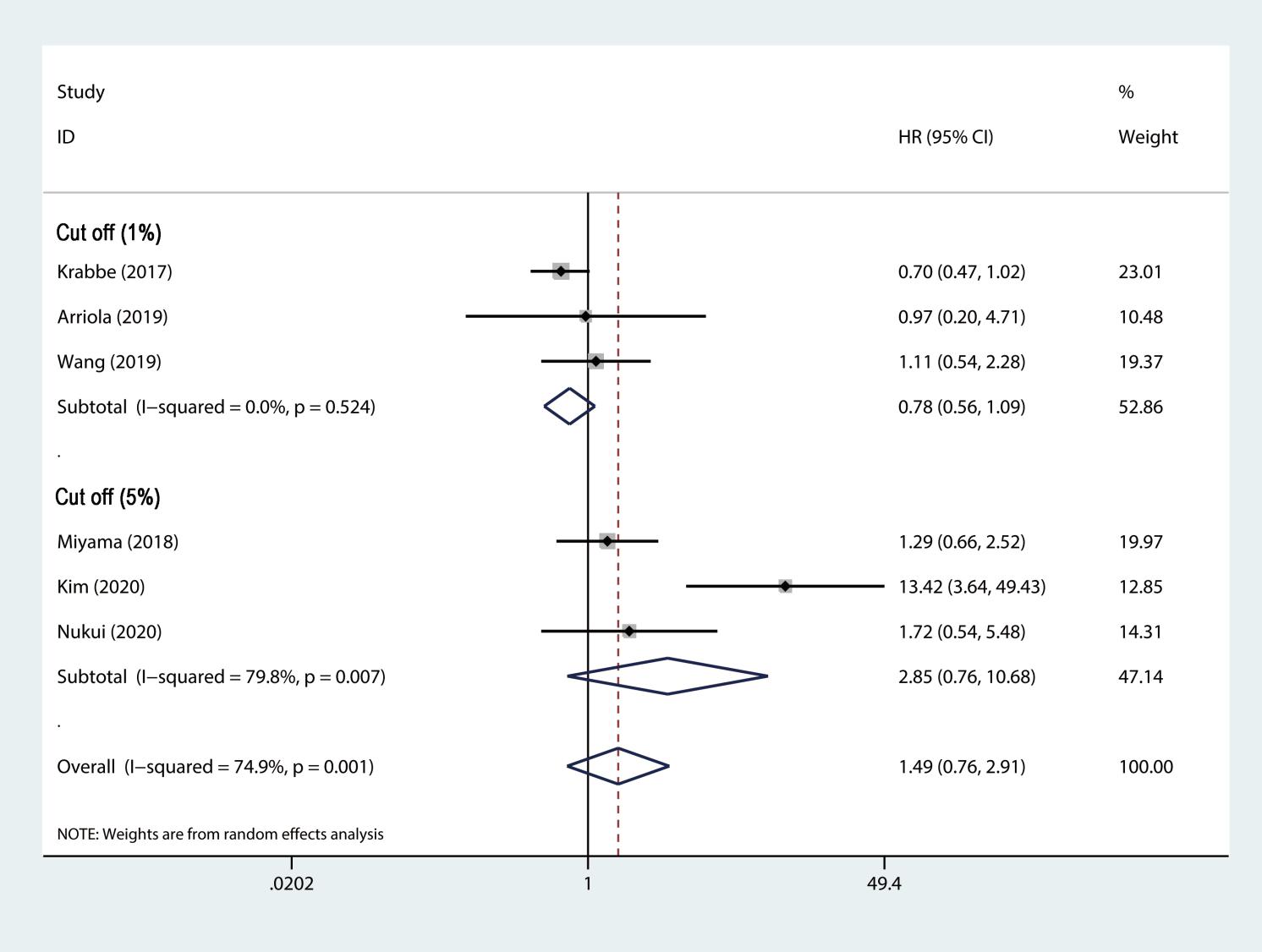


## Supplementary Figure 18. Subgroup analysis between PD-L1 expression and OS by cut-off value for PD-L1.

HR: hazards ratio; CI: confidence interval.


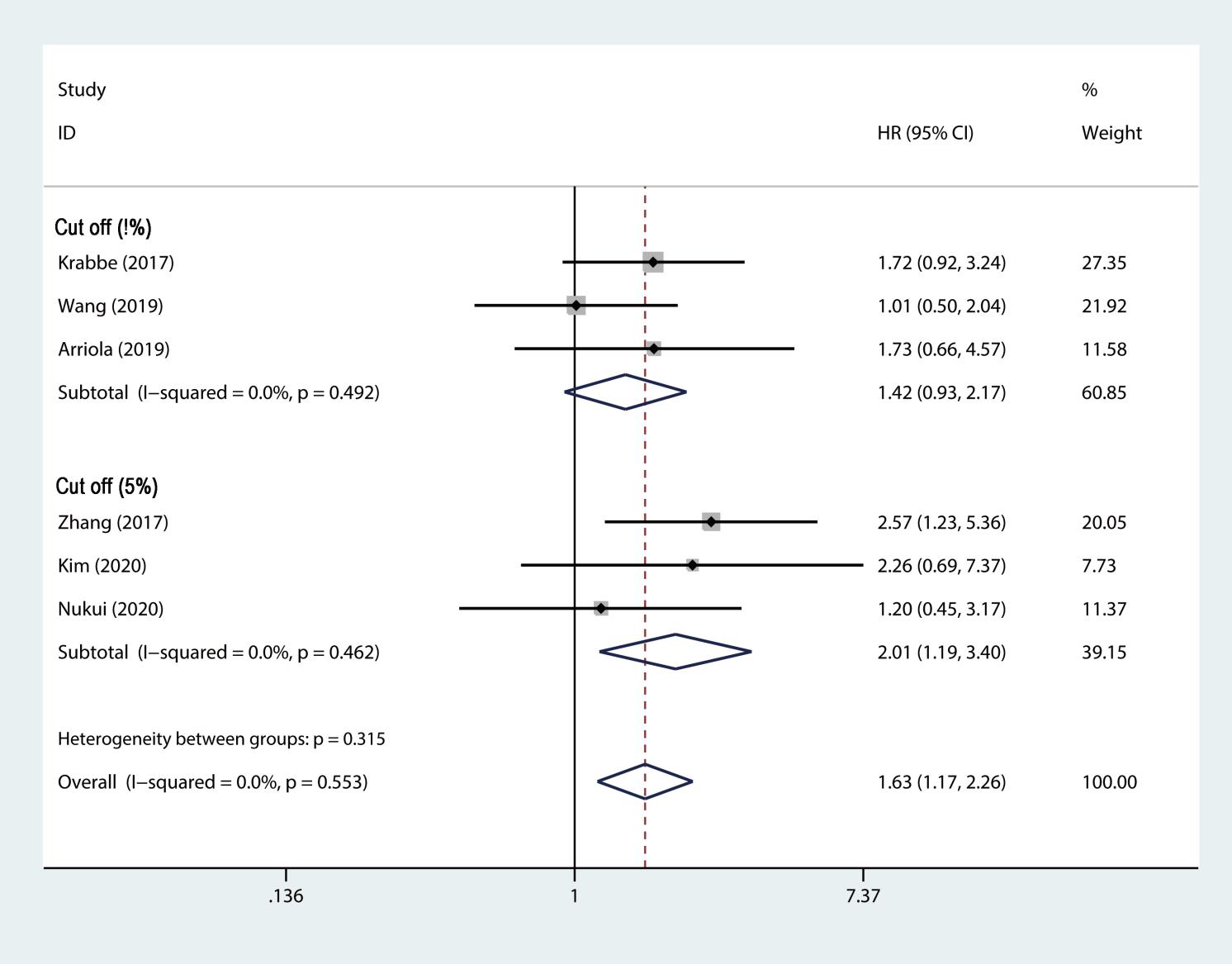


## Supplementary Figure 19. Subgroup analysis between PD-L1 expression and CSS by cut-off value for PD-L1.

HR: hazards ratio; CI: confidence interval.
